# Role of Notch signaling pathway in joint homeostasis and osteoarthritis

**DOI:** 10.3389/fbioe.2026.1861292

**Published:** 2026-09-07

**Authors:** Xiaohan Wei, Bowen Guan, Xinyi He, Songsong Zhu, Ruiye Bi

**Affiliations:** 1 State Key Laboratory of Oral Diseases, Department of Orthognathic and TMJ Surgery, National Clinical Research Center for Oral Diseases, West China Hospital of Stomatology, Sichuan University, Chengdu, China; 2 Stomatology Hospital, School of Stomatology, Zhejiang University School of Medicine, Hangzhou, Zhejiang, China

**Keywords:** chondrocyte homeostasis, extracellular matrix, Notch signaling pathway, osteoarthritis, stem cell therapy

## Abstract

The Notch signaling pathway is pivotal in joint homeostasis and the pathogenesis of osteoarthritis (OA). Under physiological conditions, transient or physiological Notch signaling maintains cartilage matrix synthesis to preserve joint function. Under pathological conditions, persistent or excessive activation of Notch signaling suppresses the expression of chondrogenic genes and induces the production of catabolic factors, thereby driving OA progression. From a cellular perspective, the Notch signaling pathway exerts crucial regulatory role in the functions of various resident cell types within the joint. For instance, it regulates the differentiation and maturation of chondrocytes, influences the chondrogenic differentiation process of mesenchymal stem cells (MSCs), and modulates the phenotype of fibroblast-like synoviocytes (FLSs). At the level of the extracellular cartilage microenvironment, the Notch signaling pathway participates in extracellular matrix (ECM) homeostasis imbalance and inflammatory factor activation in OA by regulating the expression of downstream genes. Furthermore, Notch controls chondrocyte hypertrophic degeneration through extensive molecular crosstalk with the TGF-β/BMP, Wnt/β-catenin, NF-κB, and Hippo-YAP pathways. Beyond cartilage, Notch plays a crucial role in promoting neurovascular invasion at the osteochondral junction and abnormal subchondral bone remodeling, which directly contributes to joint pain and structural failure. Many Notch-targeted approaches, such as pharmacological inhibitors, RNA-based therapies, and molecular interventions targeting ligands and downstream effectors, have been investigated because existing treatments are unable to stop the progression of OA. Simultaneously, stem cell-based approaches use precise Notch modulation to improve cartilage repair and chondrogenic differentiation. However, systemic administration of small-molecule inhibitors raises concerns about off-target effects and delivery inefficiency in avascular cartilage, while conventional stem cell injections frequently encounter problems like phenotypic instability and limited durability. In conclusion, in-depth studies on the Notch signaling pathway in OA not only clarify the pathogenesis of OA but also lay a theoretical and experimental foundation for the development of innovative therapeutic strategies.

## 1 Introduction

The structural integrity of human joints, which are mainly composed of cartilage, synovium, and subchondral bone, is the basis for their functional realization. These three components are structurally interrelated and functionally coordinated to jointly maintain the physiological state of the joint. Articular cartilage is composed mainly of hyaline cartilage, which consists of chondrocytes and an extensive amount of extracellular matrix (ECM). The ECM is formed mainly by the cross-linking of water, collagen, elastic fibers, and aggrecan (ACAN) molecules (Sophia Fox et al., 2009; Akkiraju and Nohe, 2015). Articular cartilage primarily consists of type II collagen (COL2A1) and is structurally divided into superficial, middle, and deep layers according to variations in cellular arrangement and collagen fiber distribution. Chondrogenesis and chondrocyte differentiation start with the aggregation of mesenchymal cells (MSCs), followed by the gradual maturation of chondrocytes through proliferation, hypertrophy, and matrix secretion (Fujimaki and Tezuka, 2006). However, cartilage lacks blood vessels, lymphatics, and nerves, resulting in an extremely weak repair ability after injury. The synovium is composed of a lining layer and a sublining layer. Fibroblast-like synoviocytes (FLSs) in the lining layer secrete lubricin (PRG4) to maintain joint lubrication and regulate synovial fluid composition. The sublining layer contains FLSs, resident macrophages, blood vessels, and nerves, which can assist in weight bearing, supply energy to cartilage, and remove waste (Schaible and Schmidt, 1985). Under physiological conditions, the synovium maintains the homeostasis of synovial fluid. Although subchondral bone is a supporting structure, the blood perfusion of its vascular sinusoids meets more than 50% of the nutritional needs of cartilage. Its biomechanical properties, such as the elastic modulus, also affect the stress distribution in cartilage. An abnormal structure of subchondral bone can indirectly exacerbate cartilage damage (Imhof et al., 2000), and disruption of joint homeostasis can disrupt the balance between cartilage synthesis and degradation, triggering synovial inflammation, cartilage degeneration, and joint damage, ultimately leading to OA.

OA, a degenerative joint disorder associated with aging, is triggered by alterations in local joint mechanical loads, resulting in variations in one or more signaling pathways initiated from either the synovial tissue or the articular cartilage (Chen, 2022). Clinically, it typically occurs in the knee joint, hip joint, temporomandibular joint, or small joints (Chen, 2022). Recent longitudinal studies have indicated that early molecular and morphological alterations commence in synovial tissue, followed by changes in the articular cartilage and subchondral bone (Liao et al., 2020). Importantly, OA is the most prevalent form of arthritis. Under pathological conditions, FLSs and macrophages are activated and secrete inflammatory factors such as interleukin-1β (IL-1β) and tumor necrosis factor-α (TNF-α) (Sun and Yokota, 2002), which directly regulate the expression of genes involved in cartilage matrix metabolism, disrupting the balance between cartilage synthesis and degradation and causing synovial inflammation and cartilage degeneration (Kapoor et al., 2011). Furthermore, chondrocytes undergo hypertrophic differentiation characterized by the secretion of type X collagen (COL10A1), followed by the transformation of avascular cartilage tissue into highly vascularized bone tissue through cartilage matrix degradation and vascular infiltration (Ortega et al., 2004). Recent research suggests that vascular infiltration and neural ingrowth within the articular cartilage might be the cause of OA in a subset of patients (Zhou F. et al., 2022). Limited articular cartilage repair capacity is a factor in progressive cartilage degeneration and the development of OA after cartilage injury. Thus, extensive research has been conducted on the repair of OA-related cartilage defects, among which cell-based therapies show considerable promise.

Signaling pathway dysregulation is the primary cause of the pathological progression from early synovial lesions to articular cartilage degeneration and damage, which is caused by the synergistic effects of several processes and cell types. Among the numerous signaling pathways involved in OA regulation, the Notch signaling pathway profoundly influences OA progression. Notch has long been recognized as an ancient and highly conserved signaling pathway that is widely present in both chordates and nonchordates and is involved in regulating key processes during embryonic development, cell fate determination, proliferation, differentiation, and homeostasis *in vivo* through interactions between neighboring cells (Siebel and Lendahl, 2017). Therefore, abnormal Notch signaling may lead to pathological consequences. The Notch signaling pathway consists of three components: the Notch receptor, the ligand, and the CSL protein (also known as CBF1, RBPJ, Su(H), or Lag-1). In humans, there are four Notch receptors (NOTCH1-4) and five different Notch ligands (JAG1, JAG2, DLL1, DLL3, and DLL4). The binding of ligands to the Notch receptor induces site-specific cleavage, ultimately leading to the release of the Notch intracellular domain (NICD). The NICD then translocates to the nucleus and regulates gene expression by interacting with the CSL of transcription factors. Notch activation leads to the elevated expression of specific genes, including those in the *HES* and *HEY* transcription factor families (Zhou B. et al., 2022). Recent studies have shown that the Notch pathway is much broader and more complex than previously thought and that it also plays a key role in maintaining cartilage homeostasis.

**FIGURE 1:**
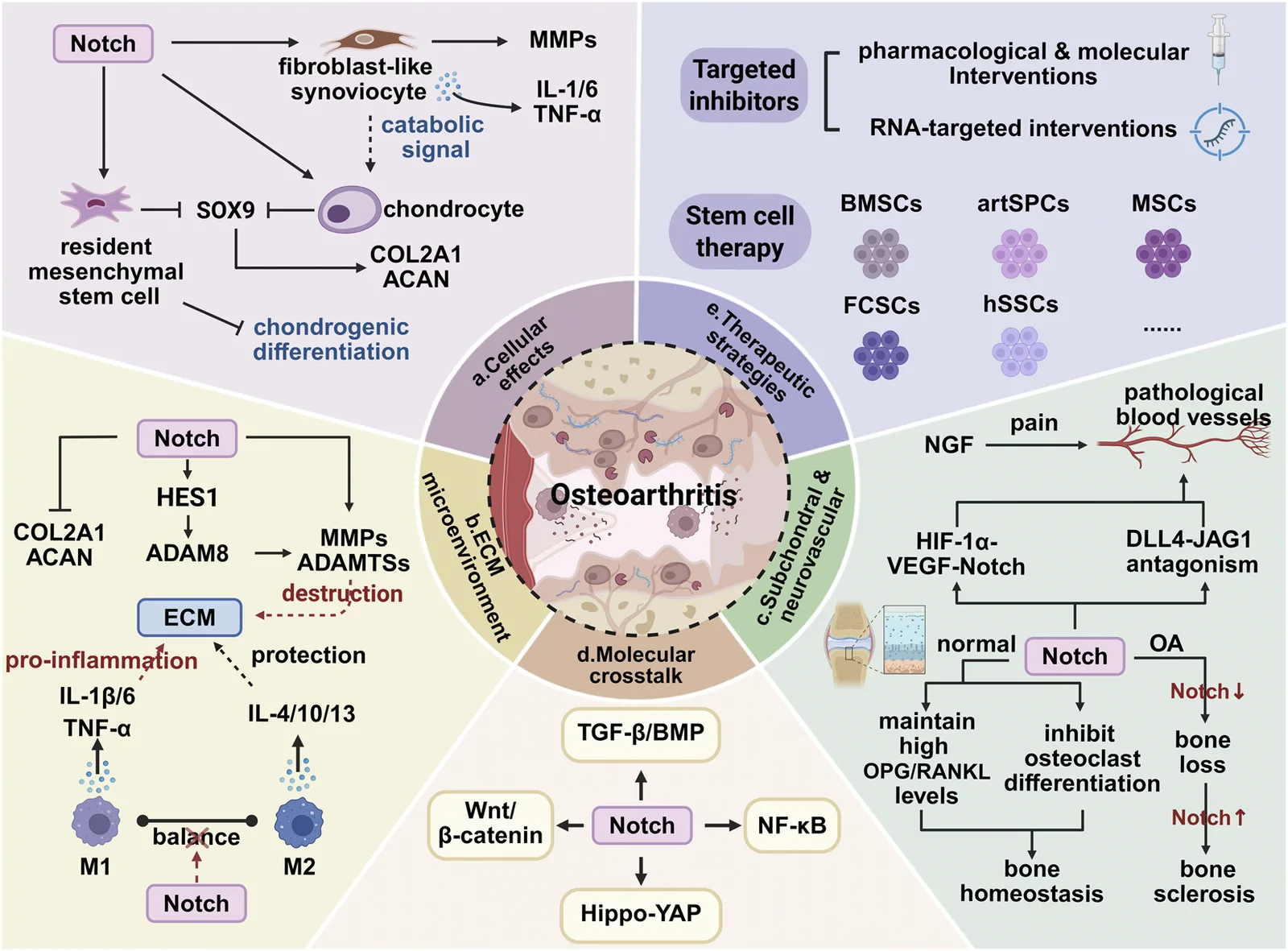
**(a)** The Notch signaling pathway significantly affects articular chondrocytes, resident mesenchymal stem cells, and fibroblast-like synoviocytes. **(b)** Overactivation of the Notch signaling pathway leads to ECM imbalance and cartilage homeostasis disruption. It can also regulate the secretion of inflammatory factors by inflammation-associated cells, and different inflammatory factors can positively or negatively regulate OA. **(c)** The Notch signaling pathway orchestrates aberrant subchondral bone remodeling and sclerosis, while interacting with neurovascular factors to drive angiogenesis and nerve ingrowth at the osteochondral junction, directly contributing to joint pain. **(d)** The Notch signaling pathway engages in extensive molecular crosstalk with core signaling cascades—including Wnt/β-catenin, TGF-β/BMP, Hippo-YAP, and NF-κB—forming a complex regulatory network that dictates chondrocyte hypertrophy, matrix degradation, and joint microenvironmental inflammation. **(e)** There are many targets in the Notch signaling pathway that can positively or negatively regulate the development of OA. Targeting these components could yield new ideas for the treatment of OA, and a variety of stem cell therapies have been gradually applied in the treatment of OA. Created in BioRender. Bi, R. (2026) https://BioRender.com/kl0bo1p.

Previous reviews mainly focused on Notch signaling in isolated cartilage or skeletal development. Nevertheless, there is still a lack of a thorough synthesis that integrates clinical translation and multi-tissue crosstalk in OA. This review offers a comprehensive organ-level framework to close this gap. We establish a direct connection between whole-joint pathology and precision therapies and dysregulated Notch signaling. In particular, we talk about how Notch controls inflammatory microenvironments and multicellular crosstalk. We also investigate its role in neurovascular co-invasion and subchondral bone remodeling. Additionally, we assess new bioengineering approaches to address important translational bottlenecks in targeted OA therapy, such as smart delivery systems and synthetic receptors (Figure 1).

## 2 Effects of the Notch signaling pathway on intra-articular cells in osteoarthritis

### 2.1 Articular chondrocytes

#### 2.1.1 Mechanism of the Notch signaling pathway in articular chondrocytes

In the extracellular domain of the Notch signaling pathway, ligands and receptors associated with cartilage homeostasis exist. Notch signaling is transmitted through a receptor‒ligand binding mechanism between adjacent cells. Three major Notch receptors are involved in the generation and differentiation of chondrocytes in both humans and mice. The Notch1 receptor is highly expressed in mouse mesenchyme condensation, preembryonic proliferation, and the hypertrophic zone and superficial layer of articular cartilage (Watanabe et al., 2003). Notch1 is expressed early in cartilage generation, and its expression decreases as cells mature (Fujimaki et al., 2006). Therefore, under physiological conditions, transient Notch signaling is crucial for maintaining cartilage matrix synthesis and joint function. The Notch2 receptor is broadly distributed throughout proliferating, prehypertrophic, and hypertrophic chondrocytes in articular cartilage and the epiphyseal growth plate (Hayes et al., 2003). Notch2 is a regulator of the activation of Notch1 and Notch3 and plays a major role in growth plate cartilage cell hypertrophy (Tu et al., 2012). Other studies have shown that Notch2 is a genetic modifier of Jagged1 at initiation; it is also tightly localized to downstream targets of Notch signaling, such as *Hes1* and *Hey1* (Dong et al., 2010). Notch3 is found mainly in mature chondrocytes and highly differentiated articular discs and can delay the cell cycle (Hayes et al., 2003). There are two main classes of Notch ligands, DLL and JAG, among which JAG1 and DLL1 are related mainly to cartilage growth and differentiation. Both ligands are expressed in the all layers of cartilage. JAG1 is a crucial factor in initiating the transmission of the Notch signaling axis (Kurenkova et al., 2023), primarily by mediating lateral induction to coordinate cellular behavior and promote uniform lineage commitment within local progenitor populations (Cai et al., 2026). Moreover, DLL acts as a negative regulator of chondrogenesis; in particular, DLL1 can inhibit mRNA expression related to the extracellular matrix and inhibit the transition from prehypertrophic to hypertrophic chondrocytes (Crowe et al., 1999).

The intracellular transduction process of Notch signaling is related to chondrocyte homeostasis. After binding to DLL or JAG, a disintegrin and metalloproteinase (ADAM) located on the membrane is activated, causing Notch receptor cleavage and the formation of the Notch cell-extracellular cleavage (NEXT) fragment (Dietrich et al., 2022). The γ-secretase enzyme subsequently acts on the NEXT fragment (Engin and Lee, 2010), cleaving the NEXT fragment to generate the NICD. Studies have shown that the expression of NICD1 under normal conditions can promote the expression of *Sox9*, *Col2a1*, and *Acan* while reducing the expression of the degrading factor *matrix metalloproteinase 13 (MMP13)*, thereby maintaining environmental homeostasis (Haller et al., 2012).

The Notch signaling pathway modulates cellular homeostasis by influencing the expression of downstream genes and interacting with other pathways. In the classical Notch pathway, the NICD is transferred to the nucleus of the cell, where it binds to the transcription factor recombination signal binding protein for immunoglobulin kappa J region gene (RBPJ) and the transcriptional activator mastermind-like 1 (MAML1). After MAML1 recruits the histone acetyltransferase p300, the NICD, RBPJ and MAML1 complex promotes the expression of the *Hes* and *Hey* families of basic helix-loop-helix transcription factors, which in turn suppresses the expression of downstream target genes (Engin and Lee, 2010).

#### 2.1.2 Notch signaling mediates articular chondrocyte hypertrophy and apoptosis in osteoarthritis

The expression levels of *NOTCH1*, *NOTCH2*, *JAG1*, *DLL1*, *HES1* and *HES5* are elevated in patients with degenerative OA, with the most significant increases in *NOTCH1*, *JAG1*, and *HES1* (Karlsson et al., 2008), which are positively correlated with the severity of OA. Mechanistically, rather than being an epiphenomenon, this persistent pathological Notch activation directly enforces a pathogenic cell fate by functionally orchestrating chondrocyte hypertrophy and apoptosis. ACAN and COL2A1 are markers of mature chondrocytes, whereas COL10A1, MMP13, runt-related transcription factor 2 (RUNX2) and alkaline phosphatase (ALPL) are markers of hypertrophic chondrocytes and OA (Mazor et al., 2022). During the process of chondrocyte generation and differentiation, Notch functions upstream of *Sox9* to suppress its expression (Chen et al., 2014). Sox9 is an important factor that regulates the development of chondrocytes and promotes the expression of *Col2a1*, *Col9a1*, and *Acan*, thereby promoting the proliferation of chondrocytes (Kohn et al., 2015). *NOTCH1* and *NOTCH2* expression is upregulated in OA chondrocytes. Activated Notch signaling results in the formation of a transcriptional activator through binding to RBPJ protein, thereby inducing the regulatory function of HES/HEY family proteins (Grogan et al., 2008). HES1 and HEY1 function at the *SOX9* binding site within the *COL2A1* enhancer to inhibit *SOX9*-mediated *COL2A1* transcriptional activation (Haller et al., 2012). HES5 functions as a repressor of *SOX9* transcription by binding to the upstream promoter region of *SOX9*, resulting in a negative correlation with its expression (Karlsson et al., 2010). Therefore, NOTCH1 overexpression blocks the transcription of *SOX9* and *COL2A1* by inducing the expression of *HES1*, thereby inhibiting chondrocyte maturation (Grogan et al., 2008). Moreover, the expression of *Notch2* suppresses the tendency of Sox9 protein expression to increase with chondrocyte maturation and proliferation, leading to the hypertrophy of chondrocytes in the proliferative phase and their eventual mineralization (Mead and Yutzey, 2009). In addition to Sox9, a crucial factor, Runx2, is also highly important; it serves as a target for regulating chondrocyte differentiation and apoptosis. After the cessation of *Sox9* expression, *Runx2* expression is initiated (Yamashita et al., 2009). Notch also functions upstream of *Runx2*. RBPJ increases *Runx2* promoter transcriptional activity (Wang et al., 2010). Notch-mediated Runx2 activation regulates MMP13 expression (Xiao et al., 2019), facilitates *Col2a1* degradation, and modulates *Col10a1* expression in hypertrophic chondrocytes (Ding et al., 2012), thereby promoting chondrocyte hypertrophy and terminal differentiation. Thus, as the severity of OA increases, the mRNA levels of *SOX9*, *COL2A1* and *ACAN* gradually decrease, while the expression of hypertrophic chondrocyte markers such as *COL10*, *MMP13* and *RUNX2* increases (Zhong et al., 2016).

Furthermore, Notch is overexpressed in OA cartilage, in contrast to healthy cartilage (Cao et al., 2023). While prolonged pathological Notch activation encourages chondrocyte senescence and the development of OA, transient physiological Notch activation is essential for progenitor cell expansion and early tissue patterning (Liu et al., 2015; Hosaka et al., 2013). The temporal dynamics of NICD nuclear accumulation and the makeup of transcriptional complexes it forms provide the mechanistic foundation for the conflicting functions of Notch signaling. In physiological settings, chondrocyte proliferation and the expression of master chondrogenic regulators are maintained by either baseline repressor expression or transient, RBPJ-dependent Notch activation (Liu et al., 2016; Mirando et al., 2013). On the other hand, high-amplitude, persistent NICD accumulation is caused by pathological conditions in OA. We conclude that this sustained activation in OA drives prolonged *Hes1/Hey1* occupancy at the regulatory regions of master chondrogenic regulators, such as Sox9 and Col2a1, by drawing an analogy to the fundamental paradigm where oscillatory *versus* sustained signaling dynamics molecularly dictate divergent cell fates (Nandagopal et al., 2018; Imayoshi et al., 2013). This extended binding recruits Groucho/TLE corepressors through the conserved C-terminal WRPW domain of Hes1, since persistent repressor occupancy often acts as a scaffold for chromatin remodeling (Majidinia et al., 2018; Oldershaw and Hardingham, 2010). Through its glycine/proline-rich (GP) domain, this TLE scaffold directly interacts with and attracts the histone deacetylase Rpd3, the mammalian HDAC1/2 homolog (Majidinia et al., 2018). The transition from active histone deacetylation to persistent DNA hypermethylation is then facilitated by the physical association of the ATRX-homology domain of the scaffolded DNA methyltransferases, namely, DNMT3A, with HDAC1 (Pakvasa et al., 2021). This multi-protein assembly creates a feed-forward repressive chromatin loop that prevents master transcription factors from binding, permanently silencing anabolic genes such as *Sox9* and *Col2a1* (Mamachan et al., 2024). At the same time, promoter site-specific CpG demethylation permits the aberrant activation of catabolic matrix-degrading enzymes such as *MMP3*, *MMP9*, *MMP13*, and *A Disintegrin and Metalloproteinase with Thrombospondin Motif 4(ADAMTS4)* (Grogan et al., 2009; Choi et al., 2016), ultimately leading to hypertrophic and catabolic cell fates in chondrocytes.

### 2.2 Resident mesenchymal stem cells (R-MSCs)

Studies have shown that the Notch signaling pathway is involved in the chondrogenic differentiation of resident mesenchymal stem cells (R-MSCs). In joints, both chondroprogenitor cells and MSCs express Notch-related signaling molecules (Majidinia et al., 2018), with NOTCH2 being the most prominent. MSC-derived articular chondrocytes express markers associated with chondrogenesis, such as the chondrogenic transcription factor *SOX9* and ECM-related genes, which include *COL2*, *COL9*, *ACAN*, *DCN*, and *cartilage oligomeric matrix protein (COMP)* (Oldershaw and Hardingham, 2010).

Similar to its effects on chondrocytes, Notch signaling represses the chondrogenic differentiation of R-MSCs through Hes1/Hey1-mediated inhibition of the *Sox9* enhancer. Moreover, *Col2a1* expression is also repressed, as *Sox9* is coexpressed with *Col2a1* (Pakvasa et al., 2021). In the early stages of cartilage formation associated with MSCs, *Jagged1* gene expression is transiently increased, which affects the Notch signaling pathway, and Hes1/Hey1 are rapidly activated but for a limited duration; therefore, *Jagged1* expression is stopped before cartilage formation begins (Oldershaw and Hardingham, 2010). However, after cartilage formation begins, the overexpression of *JAG1* leads to continuous Notch signaling, interrupting the expression of the *SOX9* and *COL2A1* promoters in MSCs (Mamachan et al., 2024) and blocking cartilage formation. Thusduring the early stages of differentiation, temporary activation of the Jagged1-mediated Notch signaling pathway is needed, whereas after the onset of differentiation, the Notch signaling pathway inhibits the chondrogenic differentiation of R-MSCs, and inhibition of the Notch signaling pathway promotes chondrogenesis (Kurenkova et al., 2023).

Notably, similar mechanisms have been found in other sources of stem cells within articular cartilage. For example, chondrocyte progenitor/stem cells (CPCs) (Grogan et al., 2009) and human embryonic cartilage-derived progenitor cells (FCPCs) (Choi et al., 2016) at the site of damage are regulated by Jagged1/Notch signaling. Fibrochondral stem cells (FCSCs) located in the temporomandibular joint (TMJ) condyle are associated with the Notch signaling pathway during TMJ morphogenesis (Bi et al., 2023), and the expression of Notch receptors and ligands is upregulated in temporomandibular joint osteoarthritis (TMJOA) to promote the conversion of cartilage to bone.

Therefore, the Notch signaling pathway plays an important role in the effects of R-MSCs in OA, suggesting that inhibition of Notch signaling may be a novel strategy for the treatment of TMJOA.

### 2.3 Fibroblast-like synoviocytes (FLSs)

Synoviocyte proliferation and synovitis are among the main features of OA. Single-cell RNA-seq data revealed that *JAG1* and *NOTCH3* are expressed by all FLSs subpopulations and that NOTCH1/2 and its ligands JAG2 and DLL1/4 are important Notch signaling molecules in FLSs (Wei et al., 2020). This receptor-specific dysregulation also plays a profound role in synovial tissue integrity; single-cell transcriptomic trajectories demonstrate that a gain of NOTCH2 function fundamentally disrupts the core molecular identity of articular and fibroblast-like synoviocytes, indicating that normal NOTCH2 regulation is essential for maintaining the distinct transcription profiles of synovial lining cells (Canalis et al., 2025). Furthermore, TNF-α increases the expression of the NOTCH-related cleavage enzymes *ADAM10/ADAM17*, the transcription factor MAML2 and the target genes *MYC/HES4* in FLSs (Zack et al., 2024). Several studies have confirmed that activated FLSs in OA secrete cytokines such as IL-1, TNF-α and IL-6 and activate various cellular signaling pathways, including the Notch pathway, the IL-6/STAT3 (signal transducer and activator of transcription 3) signaling pathway and the Toll-like receptor surface receptor signaling pathway (Sun et al., 2014), as well as catabolic signals to articular chondrocytes, thereby stimulating articular chondrocytes and causing damage to cartilage. In addition, researchers have experimentally blocked the Notch ligand Dll1 and interfered with the binding of Dll1 to Notch2, which is expressed above FLSs, thereby decreasing the production of IL-6 and MMP3 and reducing the negative effects on articular cartilage homeostasis (Sekine et al., 2014).

**FIGURE 2:**
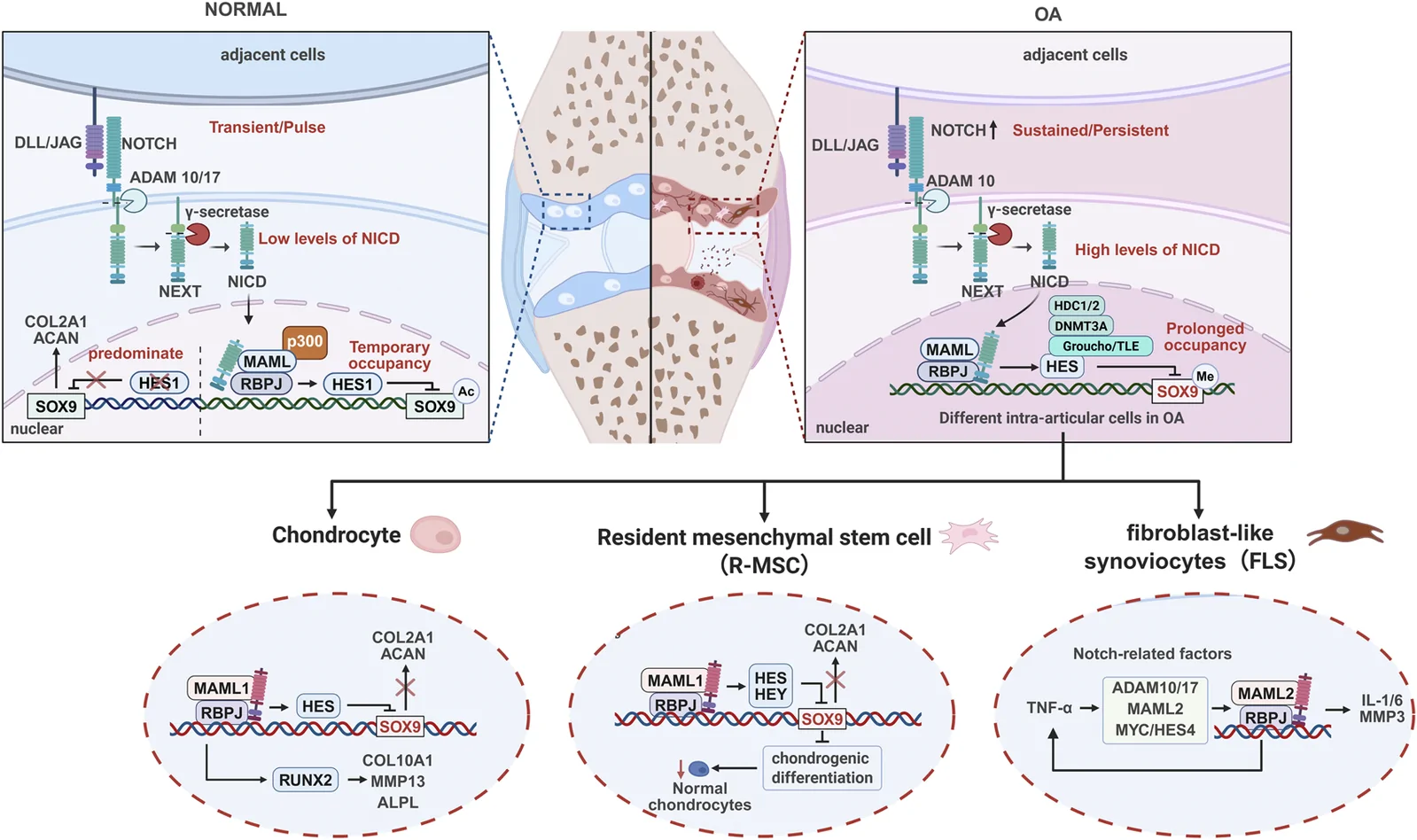
Molecular mechanisms and cell-type-specific downstream cascades of Notch signaling in OA. Physiologically, transient Notch activation maintains low nuclear NICD levels, enabling SOX9-driven transcription of *COL2A1* and *ACAN*. In OA, sustained high-amplitude NICD accumulation recruits corepressors (Groucho/TLE, HDAC1/2, DNMT3A) to form a repressive chromatin state, permanently silencing anabolic chondrogenic genes. Downstream, persistent Notch activation disrupts intra-articular homeostasis across three key cell types: driving chondrocyte hypertrophy (COL10A1, MMP13) and apoptosis via HES1/HEY1-mediated SOX9 inhibition; blocking chondrogenic differentiation in R-MSCs; and triggering TNF-α-induced ADAM10/17 and MAML2 in synovial FLSs to promote IL-1/6 and MMP3 secretion, fueling joint degradation. Created in BioRender. Bi, R. (2026) https://BioRender.com/u2vx2wf.

In conclusion, the Notch signaling pathway affects various types of cells in OA by activating different downstream pathways in them (Figure 2).

## 3 Effects of the Notch signaling pathway on the cartilaginous extracellular microenvironment in osteoarthritis

### 3.1 Effects of Notch signaling on extracellular matrix homeostasis in osteoarthritis

Under physiological conditions, joints and articular chondrocytes maintain a dynamic balance between the synthesis and degradation of ECM components. Normal articular cartilage is composed primarily of ECM rich in COL2A1 and ACAN. In OA, these proteins are degraded by specific collagenases (including MMP13, MMP9 and MMP3) and aggrecanases (including ADAMTS4 and ADAMTS5) (Heinegård and Saxne, 2011).

**FIGURE 3:**
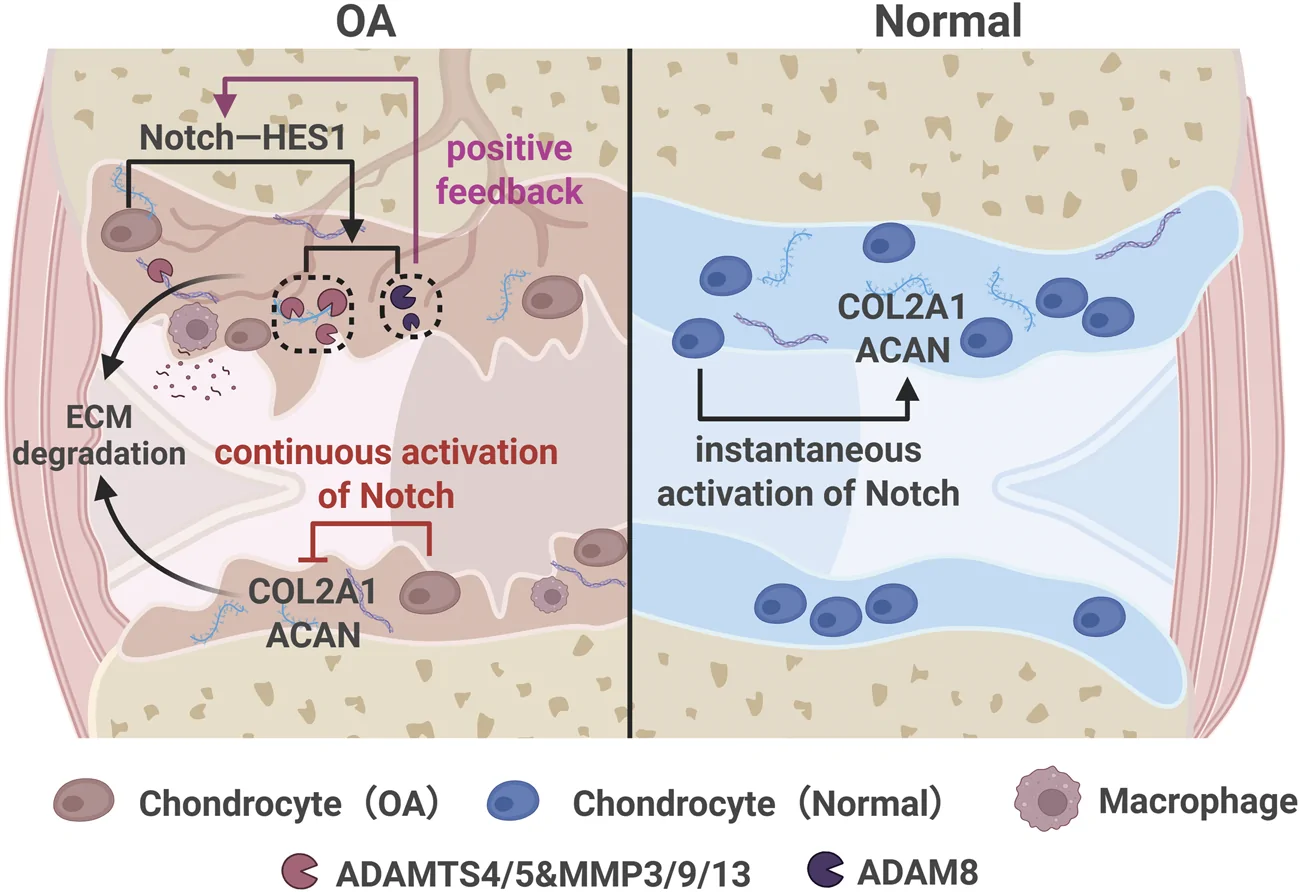
Notch signaling disrupts articular cartilage ECM homeostasis in OA. Under physiological conditions, joint and articular chondrocytes maintain a dynamic balance between the synthesis and degradation of ECM components. Normal articular cartilage is composed mainly of ECM enriched in COL2A1 and ACAN. Notch plays a dual role in the maintenance of articular cartilage and OA. In OA, activation of the Notch–HES1 system accelerates the degradation of ECM by MMPs, ADAMTSs, and ADAMs. However, transient Notch signaling activation leads to increased cartilage ECM synthesis. Created in BioRender. Bi, R. (2026) https://BioRender.com/1uum2ny.

MMP13 levels are elevated in OA cartilage, and MMP13 plays a central role in the irreversible degradation of COL2A1 in OA; thus, decreased anabolic and increased catabolic activity in chondrocytes is associated with MMP13 production (Rim et al., 2020). Additionally, in the context of OA, Hes1 can be shifted to an activated state through cofactors to directly upregulate the transcription of key catabolic enzymes, specifically *ADAMTS5* and *MMP13*, thereby exacerbating ECM degradation (Kohn et al., 2012). Previous studies have shown that Notch signaling can affect the homeostasis of the ECM in articular cartilage under OA conditions and that the upregulation of NOTCH expression can lead to an imbalance in the ECM that accelerates OA progression (Liu et al., 2023). However, another study revealed that NOTCH plays a dual role in maintaining joint cartilage and OA. The persistent activation of Notch signaling suppresses chondrogenic genes but promotes the expression of cartilage-associated proteases (MMPs and ADAMTSs), fibrillating collagen, inflammatory factors including IL-6, and many signaling pathways (tyrosine kinase, GPCR and NO signaling pathways); this also leads to severe, early and progressive OA-like lesions. In contrast, transient Notch activation leads to increased cartilage ECM synthesis and joint maintenance only under physiological conditions (Liu et al., 2015). In addition to MMPs and ADAMTSs, the ADAMs family is thought to be involved in mediating ECM degradation in OA (Liu et al., 2015). For example, *ADAM10* expression has been found to be significantly increased during OA progression and not only stimulated by the inflammatory factor IL-1 but also associated with cartilage degeneration. *ADAM9*, *ADAM12*, *ADAM15*, *ADAM19*, and *ADAM23* levels have also been shown to be increased in human OA cartilage (Chubinska et al., 2001; Isozaki et al., 2013; Swingler et al., 2009). A Notch-Hes1-ADAM8 regulatory axis has been identified in OA chondrocytes. These results indicate that Hes1 regulates *ADAM8* expression and promotes increased EGF ligand levels. EGF subsequently activates ERK1/2 and NF-κB to promote *MMP9* gene expression, which in turn promotes ECM degradation and OA progression (Duan et al., 2019). ADAM8 overexpression stimulates the expression of *Notch1* and *Hes1*, suggesting that ADAM8 can transactivate the Notch signaling pathway. Thus, a Notch-ADAM8 positive feedback loop in articular chondrocytes is involved in cartilage damage in OA. Therefore, Notch signaling disrupts articular cartilage ECM homeostasis in OA (Figure 3).

### 3.2 Effects of Notch signaling on the inflammatory microenvironment in osteoarthritis

Inflammatory cytokines act within joints and have primarily destructive effects on articular cartilage. These effects are multilayered and include not only the induction of chondrocyte senescence and apoptosis (Goldring et al., 2008) but also a reduction in the synthesis of key ECM components, such as proteoglycans, ACAN and COL1A1 (Wojdasiewicz et al., 2014). The mRNA levels of *IL-1β*, *IL-2*, *IL-12*, *IL-17*, *IL-18*, *TNF-α*, *TNF-β* and *IFN-γ* in the synovial fluid of patients with OA are significantly greater than those in the synovial fluid of normal subjects (Vernal et al., 2008). Together with IL-1β, TNF-α is considered a key inflammatory cytokine involved in OA. IL-1β and TNF-α downregulate the synthesis of major ECM components by inhibiting the activity of chondrocytes (Saklatvala, 1986). In addition, IL-1β and TNF stimulate chondrocytes to release several protein hydrolases, including MMPs, such as MMP1, MMP3, MMP13 and ADAMTS4 (Lefebvre et al., 1990) (Tortorella et al., 2001). In most cases, the role of TNF-α coincides with that of IL-1β, and there is clear synergy. IL-1β and TNF also expand and perpetuate the OA disease process by inducing the production of proinflammatory cytokines (e.g., IL-6, PGE, NO, IL-8, and CCL-5). One of these cytokines, IL-6, is also a key cytokine that synergizes with IL-1β and TNF-α, leading to a decrease in the production of COL2A1 and an increase in the production of MMPs (Sui et al., 2009). In addition, IL-4, IL-10, and IL-13 are important anti-inflammatory cytokines involved in the pathogenesis of OA.

The interaction between Notch and inflammation constitutes a bidirectional amplification system: Notch reinforces the inflammatory response, while inflammatory signals in turn trigger Notch activation. Notch activation in OA has been shown to induce the expression and activation of degradative proteases and inflammatory cytokines, ultimately leading to cartilage damage (Hosaka et al., 2013). Notch signaling leads to the amplification of macrophage-dependent inflammatory responses by enhancing NF-κB signaling. Previous studies have shown that Notch1 promotes NF-κB translocation into the nucleus and binding to DNA by increasing the phosphorylation of the IκB kinase α/β complex and the expression of several NF-κB family members (Monsalve et al., 2009). It promotes the expression of genes related to the inflammatory response, such as cytokines (e.g., *TNF-α* and *IL-6*) and effector enzymes (e.g., *inducible nitric oxide synthase (iNOS)*) (Palaga et al., 2007). The excessive release of these inflammatory factors disrupts joint homeostasis and promotes pathological damage to cartilage. Moreover, TNF increases the expression of the nonclassical NF-κB proteins p52 and RelB, which enhances the activation of Notch by binding to and promoting the nuclear translocation of the NICD to the *Hes1* promoter (Zhang et al., 2014). The Notch signaling pathway has also been implicated in the production of inflammatory cytokines in M1 macrophages, which are characterized by the production of inflammatory mediators in response to microbial product-mediated activation of Toll-like receptors (TLRs) and cytokines (Mosser and Edwards, 2008). Inflammation-associated molecules, such as TLR ligands, induce the expression of Notch receptors and their ligands and activate Notch signaling. RBPJ signaling controls the expression of the transcription factor IRF8, which induces downstream M1 macrophage-associated genes, thereby increasing the expression of TLR-induced prototypic M1 effector molecules, such as IL-12 and iNOS (Xu et al., 2012). In addition, the proinflammatory cascade induced by TNF-α, IL-1β, and IL-6 is closely associated with M1 macrophages and may lead to the production of prochondrolytic mediators, resulting in chondrocyte death and accelerated degradation of the cartilage matrix. In contrast, the expression of inflammatory mediators is lower in M2 macrophages. IL-10, Interleukin-1 receptor antagonist (IL1RA), C-C motif chemokine ligand 18 (CCL-18), insulin-like growth factor (IGF) and TGF-β are closely associated with M2 macrophages, protect cartilage and promote regeneration (Wang and He, 2022). Excessive Notch activation disturbs the homeostatic balance of M1 and M2 macrophages, tips the scale toward the M1 phenotype, and perturbs the immune microenvironment.

In addition to NF-κB-mediated feedback, various OA-associated inflammatory signals directly converge on the Notch pathway to initiate or potentiate its activation. Previous studies have shown that Notch1 mediates the TNF-α-induced proliferation of synoviocytes (Mosser and Edwards, 2008) and that Notch2 enhances the inflammatory response of chondrocytes to TNF-α. By inhibiting the synthesis of proteoglycans and COL2A1, TNF-α breaks down cartilage and promotes chondrocyte apoptosis. Moreover, this inflammatory factor can induce the expression of IL-1β and IL-6, leading to an increase in the expression of inflammatory pathways in chondrocytes (Canalis et al., 2023). Huang, Y et al. reported that IL-1β stimulation significantly increased the expression of *HSF1 (Heat shock transcription factor1)*, *EOGT (EGF-domain-specific O-linked N-acetylglucosamine transferase)*, *NOTCH1*, and *NICD1* in chondrocytes. HSFs, a family of DNA-binding proteins regulated by heat stress, regulate gene expression at the transcriptional level. HSF1 activates EOGT expression in response to IL-1β stimulation, and subsequently, EOGT promotes the O-glycosylation (O-GlcNAc) of NOTCH1 and activates NOTCH1 signaling, thereby exacerbating IL-1β-induced chondrocyte inflammation and injury (Huang et al., 2025). In addition, it has been shown that Notch mediates vascular endothelial growth factor and angiopoietin (VEGF/Ang2)-induced IL-6 and IL-8 expression in synovial explants. VEGF/Ang2 can increase the expression of intracellular NOTCH1 and NOTCH4 proteins, downstream target genes, and the ligand DLL4 (Gao et al., 2013).

**FIGURE 4:**
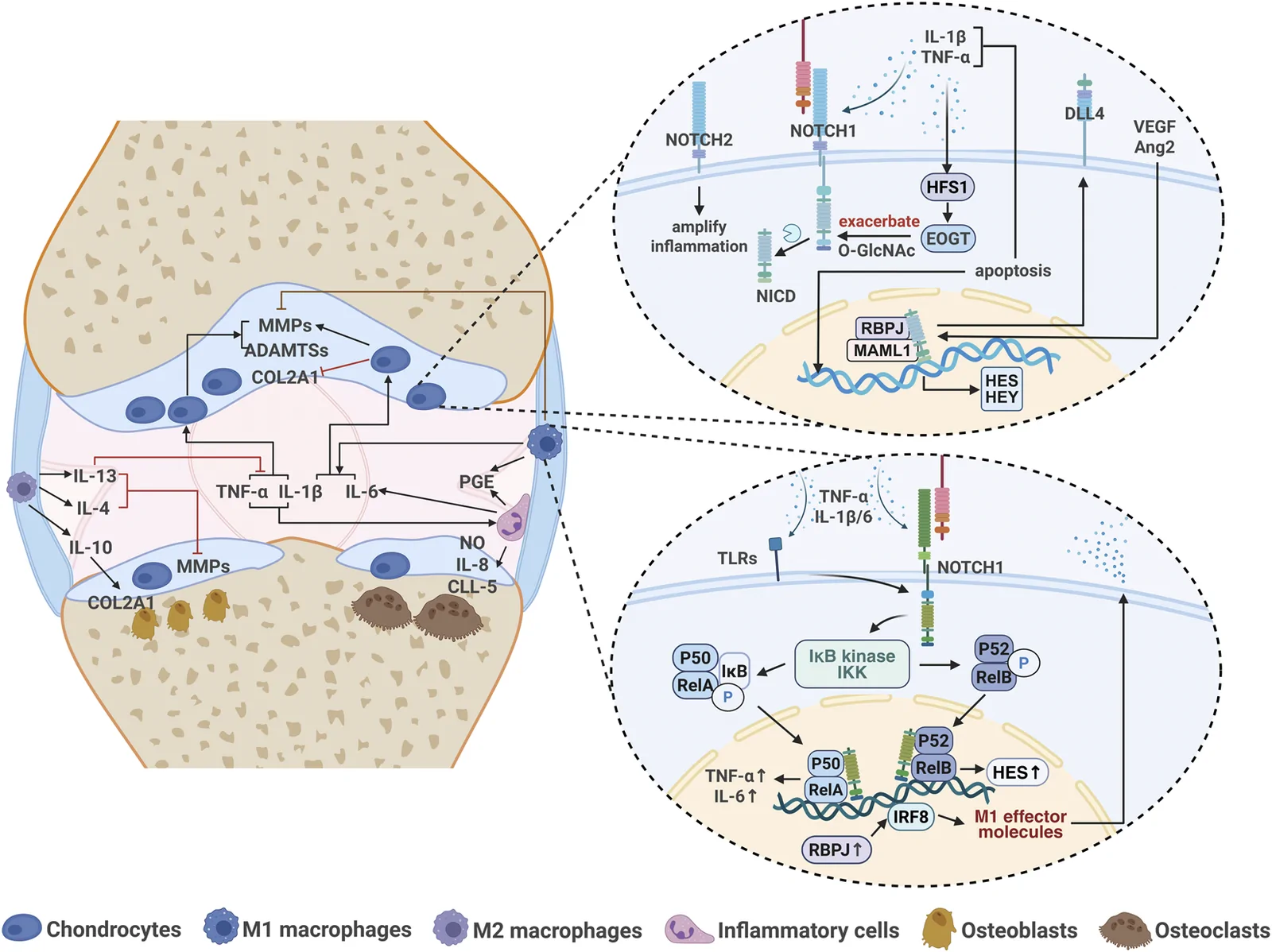
Notch signaling induces the activation of inflammatory factor expression in OA. In OA, inflammatory cytokines affect articular cartilage. Inflammatory factors, such as TNF-α and IL-1β, induce various types of degradative proteases and inhibit the production of COL2A1. Inflammatory factors activate the Notch signaling pathway, and Notch activation further induces the expression and activation of inflammatory cytokines, ultimately leading to cartilage damage. The Notch signaling pathway also regulates M1 macrophages, resulting in an imbalance between M1 and M2 macrophages, which exacerbates OA. Created in BioRender. Bi, R. (2026) https://BioRender.com/hhusxt4.

Therefore, Notch signaling induces the activation of inflammatory factor expression in OA (Figure 4).

## 4 Roles of the Notch signaling pathway in subchondral bone remodeling and neurovascular invasion in osteoarthritis

### 4.1 Notch-dependent imbalance in bone remodeling and subchondral sclerosis

An imbalance between osteoblast-mediated bone formation and osteoclast-mediated bone resorption causes subchondral bone sclerosis in advanced OA. The Notch pathway regulates this osteoblast–osteoclast coupling in a stage-dependent manner (Hu et al., 2021; Goldring et al., 2010). For instance, DLL1 promotes osteoblast progenitor proliferation while concurrently inhibiting their terminal differentiation into mature, mineralizing osteoblasts (M et al., 2017). This maturational blockade disrupts normal coupling, suppresses physiological bone turnover, and causes the excessive buildup of dense bone tissue that is indicative of sclerosis.

Under physiological conditions, canonical Notch signaling inhibits bone resorption through both direct and indirect mechanisms. Directly, Notch1 activation in myeloid progenitor cells reduces their commitment to the osteoclast lineage and their responsiveness to colony-stimulating factors, thereby suppressing osteoclast differentiation (Bai et al., 2008). Indirectly, undamaged Notch signaling in osteoblasts maintains a high osteoprotegerin (OPG)/RANKL ratio, which limits osteoclast recruitment at the tissue level (Engin et al., 2008). This homeostatic control is upset in OA due to abnormal spatiotemporal Notch activation (Zanotti and Canalis, 2016). Under pathological conditions, the overall impact varies according to the stage of the disease: in the early stages, decreased Notch signaling encourages osteoclastogenesis and bone loss; in the later stages, hyperactivation of pathways like JAG1-NOTCH2 or DLL1-NOTCH1 predominates (Wang et al., 2025). Osteoblast maturation is hampered by this hyperactivation, and osteoclast resorption is not coordinated, leading to uncoupled bone formation and resorption. Subchondral bone sclerosis eventually results from the excessive deposition of poorly mineralized woven bone caused by the ensuing dysregulated repair response.

### 4.2 Notch-driven neurovascular invasion at the osteochondral junction and joint pain

Neurovascular invasion at the osteochondral junction is driven by pathological interactions between subchondral bone remodeling, angiogenesis, and neurogenesis during the course of OA. In subchondral bone, abnormal mechanical loading triggers TGF-β1 signaling, which causes aberrant mesenchymal stem cell clustering and pathological bone remodeling (Zhen et al., 2013). By controlling the development of type H capillaries with high osteogenic potential, Notch signaling in this microenvironment links angiogenesis with bone remodeling (Peng Y. et al., 2020). Simultaneously, the HIF-1α-VEGF-Notch axis is activated by local hypoxia or inflammatory stimuli, upregulating Notch1/4 and its downstream Hes-related repressors that promote the growth of endothelial cells and neovascularization in the subchondral bone and synovium (Gao et al., 2013; Chen et al., 2019). At the ligand level, *JAG1* expression in stalk cells, altered by Fringe glycosyltransferases, opposes DLL4-Notch signaling and encourages endothelial proliferation and branching, while *DLL4* expression in endothelial tip cells limits excessive sprouting through Notch activation in neighboring cells (Benedito et al., 2009). Tip cell sprouting is altered by this DLL4-JAG1 antagonism, which also produces pathological microvascular networks that breach the avascular cartilage barrier and cross the osteochondral junction (Mapp and Walsh, 2012).

Clinical joint pain is a result of this Notch-mediated angiogenesis coupled with neurogenesis. Along with the ingrowth of pathological sensory nerve fibers along the vascular tracks from the subchondral bone, new microvessels enter the normally avascular cartilage zone as they pass through the osteochondral junction (Suri et al., 2007). Locally shared neurovascular factors are necessary for this simultaneous invasion; at the junctional zone, infiltrating macrophages and vascular endothelial cells secrete increased levels of VEGF and NGF (Walsh et al., 2010). Chronic hyperalgesia is caused by NGF, which lowers the pain threshold, sensitizes nociceptors, and directs the growth of sensory axons along pathological vessels (Mapp and Walsh, 2012). Thus, the biological basis for neurovascular co-invasion and chronic joint pain in OA is established by the mutual reinforcement of Notch-driven angiogenesis and neurotrophic factor signaling in subchondral bone.

## 5 Molecular crosstalk networks of the Notch signaling pathway in the osteoarthritis microenvironment

**FIGURE 5:**
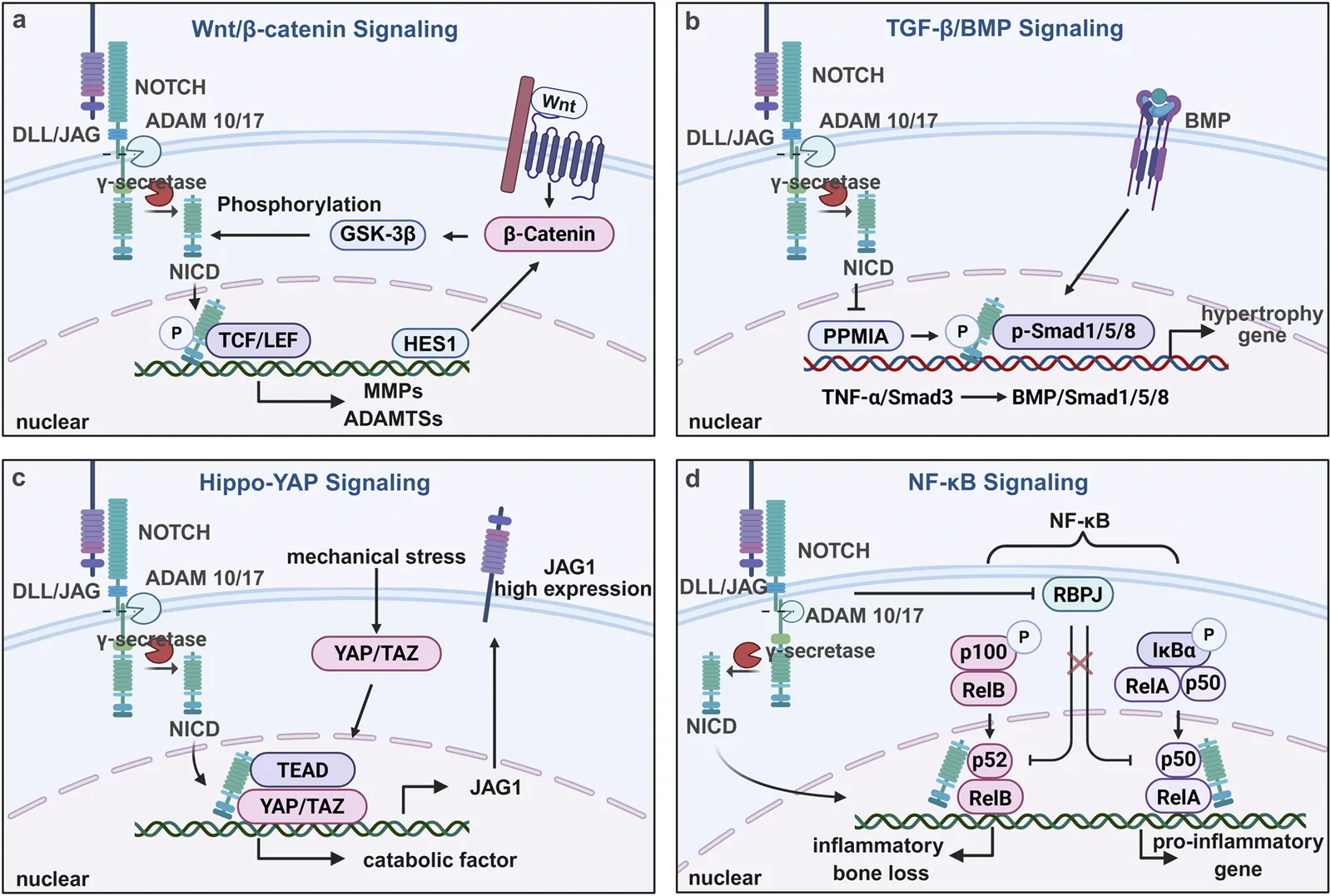
Molecular crosstalk networks of Notch signaling in OA. **(a)** Hyperactivated Notch/HES1 upregulates β-catenin to elevate MMPs and ADAMTSs, while GSK-3β phosphorylates NICD to synergistically stabilize this catabolic cascade. **(b)** Prolonged Notch activation shifts TGF-β/Smad3 homeostasis toward a BMP/Smad1/5/8-dominated pro-hypertrophic state, driving pathological terminal differentiation. **(c)** Mechanical stress triggers YAP/TAZ nuclear translocation to induce JAG1 expression and catabolic cascades. **(d)** Notch relieves RBPJ repression to activate canonical (p50/RelA) and non-canonical (p52/RelB) NF-κB pathways, fueling joint inflammation and bone loss. Created in BioRender. Bi, R. (2026) https://BioRender.com/q7k08du.

Notch signaling participates in extensive bidirectional crosstalk with several core signaling networks in the OA microenvironment, creating a macroscopic regulatory web that controls joint homeostasis and chondrocyte fate (He et al., 2020) (Figure 5).

### 5.1 Notch crosstalk with Wnt/β-catenin signaling

In articular chondrocytes, Notch and the canonical Wnt/β-catenin pathway interact primarily. Notch prevents Wnt activation under physiological conditions: the NICD physically sequesters β-catenin and limits its nuclear translocation (Kwon et al., 2011), which limits downstream osteogenic markers like ALPL and preserves the chondrocyte phenotype (Hilton et al., 2008). However, in OA, β-catenin expression is upregulated upstream of the Wnt cascade due to hyperactivated Notch/Hes1 signaling. The expression of *MMPs* and *ADAMTSs*, which break down the cartilage extracellular matrix and disturb ECM homeostasis, rises quickly as a result of Wnt/β-catenin activation (Gopalakrishnan et al., 2014). Reciprocal feedback amplifies this synergy: GSK3β phosphorylates NICD, increasing its transcriptional activity and nuclear stability (Gao et al., 2021). Furthermore, nuclear NICD becomes an independent coactivator for lymphoid enhancer-binding factor-1 (LEF-1) instead of a physiological antagonist (Fang et al., 2019; Hoeppner et al., 2009). The HMG DNA-binding domain of LEF-1 and the C-terminal TAD of NICD directly associate physically to mediate this non-canonical transactivation. Crucially, neither the traditional Wnt ligand-destruction machinery nor the canonical RBPJ complex are necessary for this interaction (Ross and Kadesch, 2001). Consequently, a catabolic shift is driven by this RBPJ-independent nuclear cooperation, as evidenced by the elevated expression of *MMPs* and *ADAMTSs*.

### 5.2 Notch crosstalk with TGF-β/BMP signaling

Additionally, Notch interacts with the TGF-β and BMP pathways in both directions. While *Smad3* loss in mice increases *Col10a1* expression and OA-like degeneration, TGF-β/Smad3 signaling suppresses chondrocyte hypertrophy and preserves cartilage integrity (Yang et al., 2001). TGF-β inhibits terminal differentiation while promoting early chondrogenesis (Feng et al., 2024). On the other hand, Notch activation increases BMP/Smad1/5/8 signaling, which leads to chondrocyte hypertrophy. According to Shang et al., NICD overexpression raises p-Smad1/5/8 levels by suppressing PPM1A, and NICD physically interacts with p-Smad1/5/8 (Shang et al., 2016). Therefore, prolonged Notch activation in OA may contribute to pathological chondrocyte terminal differentiation by shifting the balance from a homeostatic state dominated by TGF-β/Smad3 to a pro-hypertrophic state dominated by BMP/Smad1/5/8.

### 5.3 Notch crosstalk with Hippo–YAP signaling

The Hippo–YAP pathway is an essential mechanotransductive hub that controls chondrocyte phenotype and homeostatic balance by interpreting physical cues like ECM stiffness and joint loading (Han et al., 2024). Hippo-YAP and Notch are closely related: YAP/TAZ function as mechanosensors that drive *JAG1* expression through TEAD transcription factors, thereby regulating Notch signaling (Totaro et al., 2018; Jin, 2026; Tschaharganeh et al., 2013). Additionally, based on known Hippo-Notch crosstalk mechanisms in human hepatocellular carcinoma and vascular endothelial cells (Manderfield et al., 2015), we speculate that nuclear YAP/TAZ may physically bind with NICD to form a stable transcriptional complex under the aberrant mechanical strains typical of OA. The expression of catabolic factors is probably increased by this YAP-NICD axis, which successfully incorporates mechanotransduction into the Notch-mediated matrix degradation cascade.

### 5.4 Notch crosstalk with NF-κB signaling

The pathological synchronization between these two cascades is ultimately controlled by direct molecular and transcriptional crosstalk within the nucleus, as was previously discussed in relation to NF-κB signaling amplifying macrophage-dependent inflammatory responses in the joint microenvironment. RelA (p65), c-Rel, RelB, NF-κB1 (p50), and NF-κB 2 (p52) are the five members of the nuclear factor-κB (NF-κB) family that have significant roles (Osipo et al., 2008; Shin et al., 2006). This molecular crosstalk starts at the biochemical level when the IκB kinase (IKK) α/β complex is phosphorylated by the active Notch1 signaling cascade (Monsalve et al., 2009). The inhibitory IκB proteins are subsequently degraded as a result of this phosphorylation event, releasing the sequestered RelA/p50 heterodimer and promoting its nuclear translocation and DNA-binding activity to increase the expression of multiple NF-κB family members (Peng C. et al., 2020). Simultaneously, the non-canonical RelB/p52 pathway is activated without classical IκB degradation, depending instead on the controlled proteolytic processing of the p100 precursor (encoded by the *NF-κB2* gene) to produce the active p52 subunit, which then heterodimerizes with RelB for nuclear translocation (Chen et al., 2021). By promoting *Jagged1* expression, activated NF-κB increases Notch initiation; in turn, Notch avoids RBPJ-mediated repression of the *NF-κB2* gene, making NF-κB2 a direct downstream target of Notch (Oswald et al., 1998). Increases in the expression of chondrocyte inflammatory cytokines, MMPs, chemokines, and other proteins are encouraged by NF-κB, which mediates chondrocyte activation brought on by chondrocyte catabolism (Pulai et al., 2005). Furthermore, nuclear NICD engages in synergistic crosstalk with the canonical NF-κB subunit p65 (RelA) to amplify pro-inflammatory gene transcription (Cao et al., 2017), while simultaneously driving the noncanonical NF-κB pathway (p52/RelB axis) to exacerbate inflammatory bone loss in the arthritic microenvironment (Zhang et al., 2014). Notch is positioned as a key hub where mechanical stress, morphogenetic signaling, and innate immunity come together to cause cartilage degeneration because of this reciprocal feedback loop that maintains a pro-inflammatory microenvironment.

## 6 The Notch signaling pathway plays a role in articular cartilage therapy

### 6.1 Potential targets for treating osteoarthritis in the Notch signaling pathway

The currently available treatment options for OA patients include oral nonsteroidal anti-inflammatory drugs (NSAIDs), acetaminophen, and opioid analgesics, as well as intra-articular injections such as steroids, hyaluronic acid, and platelet-rich plasma. These treatment strategies can provide appropriate relief but do not prevent disease progression. In the process of studying the Notch pathway, scholars have proposed many potential therapeutic targets for OA that can promote the repair of OA cartilage injury and delay the progression of OA to a certain extent.

N-[N-(3,5-difluorophenylacetate)-L-alanyl]-(S)-phenylglycine tert-butyl ester (DAPT), a pharmacological inhibitor of Notch signaling that inhibits the expression of *MMP13*, *VEGF*, and *Hes1*, is the most commonly used inhibitor to validate Notch signaling as a clinical therapeutic target in OA (Tu et al., 2012; Zheng et al., 2026). However, there are major clinical obstacles to the translational depth of γ-secretase inhibitors like DAPT. Systemic administration of γ-secretase raises serious concerns about off-target effects and systemic toxicity, including gastrointestinal complications, because it cleaves multiple trans-membrane substrates beyond Notch receptors (Searfoss et al., 2003). Moreover, a major obstacle for these targeted inhibitors is still getting around drug delivery restrictions in avascular cartilage tissue (Rothenfluh et al., 2008).

Tables 1A, B summarize the different specific molecular nodes and compounds that researchers have discovered to more precisely modulate Notch signaling in addition to global pathway inhibitors. There are two main types of these tactics. Pharmacological and molecular interventions include CaMK2 inhibition, which prevents HES1 co-activator function, and ligand-targeted strategies like soluble JAG1 for temporary competitive inhibition and GRb1 for downregulating *Jagged1/Notch1* expression (Sun et al., 2018; Wang et al., 2015). RNA-based therapies include miRNAs and RNA interference that allow *NOTCH* components or their downstream transcription factors to be regulated at the gene level. When taken as a whole, these various strategies present viable substitutes for widespread pathway suppression, enabling a transition to focused, phenotype-specific OA treatments.

**TABLE 1:** OA therapies related to the Notch signaling pathway.

Category	Name	Function	Source
A Pharmacological and molecular interventions targeting Notch signaling in OA.			
*γ-secretase inhibitors*	DAPT	Inhibits NICD generation; suppress *MMP13*, *VEGF*, *Hes1*	(Hosaka et al., 2013)
	DBZ	Inhibits *Hes1* mRNA expression in the Notch pathway	(Huang et al., 2020)
*Ligand-targeted*	Soluble Jagged-1	Instantaneous inhibition of Notch promotes the chondrogenic differentiation of placental mesenchymal stem cells (PMSCs) and avoids the side effects of constitutive Notch inhibition	(Sun et al., 2018)
	GRb1	Suppresses the Notch signaling pathway and inhibits *MMP13* and *Jagged1* expression	(Wang et al., 2015)
*Epigenetic/co-factor modulators*	CaMK2 phosphorylation	Blocks *HES1*, downregulates the transcription of *ADAMTS5/MMP13*, and suppresses the Notch pathway	(Zieba et al., 2020)
B RNA-based therapeutics targeting Notch components in OA			
*RNA-based therapy*	YY1	Inhibits *microRNAs (miR)-140-5p* expression and enhances the downstream Jagged1/Notch pathway	(Lin et al., 2017)
	miR-9	Downregulates the expression of *Notch* and *Bcl-2-associated X protein (Bax)* and activates Bcl-2 to promote chondrocyte differentiation and regeneration	(Yu et al., 2019)
	miR-145	Reduces OA-induced chondrocyte apoptosis through the targeted inhibition on *BNIP3* and regulation of the Notch pathway	(Wang et al., 2020)
	miR-140-5p	Directly targets downstream *Jagged1* and inhibits *Notch* in CPCs	(Chen et al., 2023)
	miR-150-5p	Directly inhibits *Notch1* activation and attenuates lipopolysaccharide-induced inflammatory responses and apoptosis in macrophages	(Deng et al., 2020)
	miR-30a	Negatively regulates the 3'UTR of the Notch receptor Dll4 to promote MSC differentiation into chondrocytes	(Tian et al., 2016)
	miR-23b	Negatively regulates programmed cell death 4 (PDCD4) and decreases the phosphorylation levels of p65 and IkappaBalpha (IκBα) as well as the expression of *Notch1*, *Notch2* and *Notch3*	(Yang et al., 2018)
	Runx2 RNAi	Decreases the regulatory sensitivity of Notch to *MMP13* expression	(Xiao et al., 2019)
C Stem cell-based strategies with Notch modulation			
*Stem cell therapy*	Intra-articular injection of MSCs	Directly downregulates the Notch signaling pathway	(Zhang et al., 2021)
	CPCs	miR-140 inhibits the Notch signaling pathway, induces the activation of CPCs, and enhances their proliferation and chondrogenic differentiation abilities	(Si et al., 2019)
	Human synovial mesenchymal stem cells (hSMSCs)	Jagged-1 mediated Notch signaling initiates chondrogenesis	(Oldershaw et al., 2008)
	Adipose-derived stem cells (ASCs)	miRNAs counteract immune cells when placed in an inflammatory microenvironment	(Colombini et al., 2022)
	BMSCs	miRNAs modulate inflammation and remodeling toward the restoration of tissue homeostasis	(Colombini et al., 2022)
	FCSCs	The Notch pathway can be inhibited to promote the transformation of stem cells into chondrocytes	(Ruscitto et al., 2020)
	Human adipose derived mesenchymal stem cells(hADMSCSs)	Thrombospondin 2 (TSP2) increases the expression of hADMSCS cartilage formation markers (Sox9 and Col2a1) and Notch signaling genes (*Jagged1* and *Notch3*)	(Ruscitto et al., 2020)

### 6.2 Role of Notch signaling in stem cell therapy for osteoarthritis

Previous studies have reported that stem cells from different tissue sources can be expanded *in vitro* and implanted into the body for treatment through local transplantation or injection. Stem cell therapy has emerged as a prominent approach for repairing cartilage damage, with the selection of stem cells encompassing MSCs, bone marrow mesenchymal stromal cells (BMSCs), fibrocartilage stem cells (FCSCs), stem/progenitor cells in articular cartilage (artSPCs), and human skeletal stem cells (hSSCs). The Notch signaling pathway plays a pivotal role in the chondrogenic differentiation of these stem cells. Research has demonstrated the critical involvement of the Notch signaling pathway in determining the fate of BMSCs as they differentiate into chondrocytes (Venkatesh et al., 2017), regulating FCSC chondrogenic potential (Ruscitto et al., 2020), modulating artSPC chondrogenic potential (Kurenkova et al., 2023), and influencing the chondrogenic process in hSSCs (Kobayashi et al., 2021). During the initial stage of chondrogenic differentiation in stem cells, Notch activation is essential. Research has demonstrated that the initial activation of the Notch signal alone is adequate to induce chondrogenic differentiation in stem cells (Kobayashi et al., 2021). Moreover, sustained activation of the Notch pathway suppresses the chondrogenic differentiation of stem cells. Furthermore, during the proliferation stage, the use of Notch pathway inhibitors to attenuate Notch signaling can effectively preserve the chondrogenic potential of stem cells (Mamachan et al., 2024). Therefore, we compile the potential stem cell-based therapies associated with the Notch signaling pathway in OA (Table 1C). Conventional intra-articular stem cell transplantation frequently fails to produce long-term, durable clinical outcomes despite these mechanistic insights (Occhetta et al., 2016; Chen and Tuan, 2008). There are two primary causes of this clinical translation bottleneck: First, synovial fluid shear stress and macrophage phagocytosis quickly remove unconfined stem cells injected into the joint space, resulting in low engraftment and poor retention at the lesion site (Maudens et al., 2018). Second, unconfined MSCs can change from a stable chondrogenic phenotype to an undesirable hypertrophic or degenerative fate due to their high susceptibility to pro-inflammatory cytokines in the osteoarthritic joint (Lolli et al., 2019).

Recent developments have used bioengineering techniques to improve the accuracy and effectiveness of regenerative treatments that target Notch signaling in order to get around these translational obstacles. Targeted modulation of Notch components with lower off-target risks is made possible at the molecular level by gene-editing and RNA-based therapies, such as CRISPR/Cas9 and siRNA/miRNA (DeJulius et al., 2024). Biomaterial-assisted delivery platforms, like microcarriers and functionalized scaffolds, offer spatiotemporally controlled release and structural support to direct localized chondrogenic differentiation at the tissue level (Liu et al., 2018). Engineered cell therapies, such as the Col2a1-synNotch MSC platform, provide programmable responsiveness at the cellular level by triggering therapeutic transgenes only when they detect OA-specific markers like damaged Col2a1 (Walton et al., 2025). In addition to these cell-based methods, exosome-based treatments offer a cell-free way to deliver Notch-modulating signals, avoiding the difficulties with engraftment and survival that come with live-cell transplantation (Toh et al., 2017; Cheng et al., 2022).

## 7 Conclusion

The Notch signaling pathway has emerged as a pivotal regulator of joint homeostasis and the pathogenesis of OA. Under physiological conditions, transient Notch signaling is essential for maintaining cartilage matrix synthesis and joint function. However, under pathological conditions, the persistent activation of Notch signaling disrupts cartilage homeostasis by suppressing chondrogenic genes and promoting catabolic factors, leading to OA progression. This review highlights the multifaceted roles of Notch signaling in various intra-articular cells, including articular chondrocytes, MSCs, and fibroblast-like synoviocytes. We also discuss the role of Notch signaling in regulating ECM homeostasis and the inflammatory microenvironment, and further elaborate on its critical involvement in subchondral bone remodeling and neurovascular invasion at the osteochondral junction, which directly correlates with joint pain and structural deterioration. Additionally, we summarize the extensive molecular crosstalk between Notch and other major pathways, including Wnt/β-catenin, TGF-β/BMP, and Hippo-YAP, as well as the self-sustaining amplification loop with NF-κB, all of which collectively orchestrate the degenerative cell fate in OA.

Recent advancements in understanding the Notch pathway have revealed potential therapeutic targets for OA. Pharmacological inhibitors have been developed to suppress pathological Notch activation and reduce cartilage degradation, whereas RNA-based therapies allow for gene-specific regulation of Notch components to promote chondrocyte survival and differentiation. Additional molecular interventions targeting Notch ligands, downstream effectors, and associated regulatory molecules offer new avenues for precision intervention. Simultaneously, stem cell-based therapies using various mesenchymal and progenitor cell sources use Notch modulation to improve chondrogenic differentiation and cartilage regeneration, indicating a promising avenue for disease-modifying treatment. However, several translational barriers remain. Conventional intra-articular stem cell injection frequently results in suboptimal clinical outcomes due to poor cell retention, phenotypic drift toward hypertrophic or degenerative fates during inflammation, and limited long-term engraftment. Systemic administration of pharmacological Notch inhibitors presents challenges such as off-target toxicity, as γ-secretase cleaves multiple substrates beyond Notch receptors, and low bioavailability in the avascular cartilage microenvironment.

In the future, overcoming these barriers will necessitate a paradigm shift from broad pathway inhibition to precise modulation of specific Notch nodes in a spatiotemporal controlled manner. Selective modulators, such as ligand-specific antibodies or advanced RNA therapeutics, offer greater target specificity than current γ-secretase inhibitors. Biomaterial-assisted platforms, such as functionalized scaffolds, nanoparticle carriers, and hydrogels, offer promising solutions for long-term and localized delivery in the challenging cartilage environment. Crucially, the clinical application of these delivery systems must take into account OA’s biological and structural heterogeneity. OA is not a single disease entity. Instead, it includes several clinical phenotypes, such as aging-related, inflammatory, metabolic, post-traumatic, and temporomandibular joint TMJOA. Each phenotype is determined by specific microenvironmental stressors and local cellular dynamics. As a result, standardized Notch interventions are unlikely to provide consistent efficacy for all patients. Future paradigms must shift toward personalized, phenotype-specific strategies. This requires matching the specific mode of Notch modulation to the underlying cause. For example, clinicians may require transient activation to prime stem cells or sustained inhibition to prevent hypertrophic degradation. Interventions must also consider the anatomical context, such as TMJ fibrocartilage or knee hyaline cartilage. By combining single-cell and spatial omics with smart biomaterial platforms, targeted Notch therapies can achieve localized and spatiotemporally controlled delivery while reducing systemic off-target risks. Ultimately, we envision that the integration of pharmacological, RNA-based, and stem cell strategies with advanced delivery systems will be the most effective route toward achieving durable, disease-modifying outcomes in OA.

In conclusion, in-depth studies on the Notch signaling pathway in OA not only clarify the pathogenesis of OA but also lay a theoretical and experimental foundation for the development of innovative therapeutic strategies.

## References

[B147] Akkiraju H. Nohe A. (2015). Role of chondrocytes in cartilage formation, progression of osteoarthritis and cartilage regeneration. J. Dev. Biol. 3 (4), 177–192. 10.3390/jdb3040177 27347486 PMC4916494

[B2] Bai S. Kopan R. Zou W. Hilton M. J. Ong C. t. Long F. (2008). NOTCH1 regulates osteoclastogenesis directly in osteoclast precursors and indirectly *via* osteoblast lineage cells. J. Biol. Chem. 283 (10), 6509–6518. 10.1074/jbc.M707000200 18156632

[B3] Benedito R. Roca C. Sörensen I. Adams S. Gossler A. Fruttiger M. (2009). The notch ligands Dll4 and Jagged1 have opposing effects on angiogenesis. Cell 137 (6), 1124–1135. 10.1016/j.cell.2009.03.025 19524514

[B4] Bi R. Li Q. Li H. Wang P. Fang H. Yang X. (2023). Divergent chondro/osteogenic transduction laws of fibrocartilage stem cell drive temporomandibular joint osteoarthritis in growing mice. Int. J. Oral Sci. 15 (1), 36. 10.1038/s41368-023-00240-5 37626033 PMC10457315

[B5] Cai D. Shao J. Xiao Y. (2026). Notch signalling in alveolar bone development and regeneration: a critical regulator for osteoblasts to osteocytes transitioning. Dent. Res. 1 (2), 100025. 10.1016/j.dtrs.2026.100025

[B6] Canalis E. Yu J. Singh V. Mocarska M. Schilling L. (2023). NOTCH2 sensitizes the chondrocyte to the inflammatory response of tumor necrosis factor α. J. Biol. Chem. 299 (12), 105372. 10.1016/j.jbc.2023.105372 PMC10692730 37865314

[B7] Canalis E. Guzzo R. Schilling L. Denker E. (2025). NOTCH2 disrupts the synovial fibroblast identity and the inflammatory response of epiphyseal chondrocytes. J. Biol. Chem. 301 (6), 110206. 10.1016/j.jbc.2025.110206 40345585 PMC12179613

[B8] Cao Q. Karthikeyan A. Dheen S. T. Kaur C. Ling E. A. (2017). Production of proinflammatory mediators in activated microglia is synergistically regulated by Notch-1, glycogen synthase kinase (GSK-3beta) and NF-kappaB/p65 signalling. PLoS One 12 (10), e0186764. 10.1371/journal.pone.0186764 29049420 PMC5648239

[B9] Cao H. Yang P. Liu J. Shao Y. Li H. Lai P. (2023). MYL3 protects chondrocytes from senescence by inhibiting clathrin-mediated endocytosis and activating of Notch signaling. Nat. Commun. 14 (1), 6190. 10.1038/s41467-023-41858-7 37794006 PMC10550997

[B10] Chen D. (2022). Osteoarthritis: a complicated joint disease requiring extensive studies with multiple approaches. J. Orthop. Transl. 32, 130. 10.1016/j.jot.2022.02.009 PMC9072797 35591933

[B11] Chen F. H. Tuan R. S. (2008). Mesenchymal stem cells in arthritic diseases. Arthritis Res. Ther. 10 (5), 223. 10.1186/ar2514 18947375 PMC2592798

[B12] Chen S. Lee B. H. Bae Y. (2014). Notch signaling in skeletal stem cells. Calcif. Tissue Int. 94 (1), 68–77. 10.1007/s00223-013-9773-z 23963632 PMC3947214

[B13] Chen Y. Zhao B. Zhu Y. Zhao H. Ma C. (2019). HIF-1-VEGF-Notch mediates angiogenesis in temporomandibular joint osteoarthritis. Am. J. Transl. Res. 11 (5), 2969–2982. 31217867 PMC6556632

[B14] Chen Q. Lu X. Zhang X. (2021). Noncanonical NF-κB signaling pathway in liver diseases. J. Clin. Transl. Hepatol. 9 (1), 81–89. 10.14218/jcth.2020.00063 PMC7868705 33604258

[B15] Chen Y. Liao G. Ma T. Li L. Yang J. Shen B. (2023). YY1/miR-140-5p/Jagged1/Notch axis mediates cartilage progenitor/stem cells fate reprogramming in knee osteoarthritis. Int. Immunopharmacol. 121, 110438. 10.1016/j.intimp.2023.110438 37295026

[B16] Cheng J. Sun Y. Ma Y. Ao Y. Hu X. Meng Q. (2022). Engineering of MSC-derived exosomes: a promising cell-free therapy for osteoarthritis. Membr. (Basel) 12 (8), 739. 10.3390/membranes12080739 PMC9413347 36005656

[B17] Choi W. H. Kim H. R. Lee S. J. Jeong N. Park S. R. Choi B. H. (2016). Fetal cartilage-derived cells have stem cell properties and are a highly potent cell source for cartilage regeneration. Cell Transpl. 25 (3), 449–461. 10.3727/096368915X688641 26171766

[B18] Chubinskaya S. Mikhail R. Deutsch A. Tindal M. H. (2001). ADAM-10 protein is present in human articular cartilage primarily in the membrane-bound form and is upregulated in osteoarthritis and in response to IL-1alpha in bovine nasal cartilage. J. Histochem Cytochem 49 (9), 1165–1176. 10.1177/002215540104900910 11511685

[B19] Colombini A. Libonati F. Lopa S. Ragni E. De Luca P. Zagra L. (2022). Immunomodulatory potential of secretome from cartilage cells and mesenchymal stromal cells in an arthritic context: from predictive fiction toward reality. Front. Med. (Lausanne) 9, 992386. 10.3389/fmed.2022.992386 36314003 PMC9596769

[B20] Crowe R. Zikherman J. Niswander L. (1999). Delta-1 negatively regulates the transition from prehypertrophic to hypertrophic chondrocytes during cartilage formation. Development 126 (5), 987–998. 10.1242/dev.126.5.987 9927599

[B21] DeJulius C. R. Walton B. L. Colazo J. M. d'Arcy R. Francini N. Brunger J. M. (2024). Engineering approaches for RNA-based and cell-based osteoarthritis therapies. Nat. Rev. Rheumatol. 20 (2), 81–100. 10.1038/s41584-023-01067-4 PMC11129836 38253889

[B22] Deng X. Lin Z. Zuo C. Fu Y. (2020). Upregulation of miR-150-5p alleviates LPS-induced inflammatory response and apoptosis of RAW264.7 macrophages by targeting Notch1. Open Life Sci. 15 (1), 544–552. 10.1515/biol-2020-0058 33817242 PMC7874594

[B23] Dietrich B. Haider S. Meinhardt G. Pollheimer J. Knöfler M. (2022). WNT and NOTCH signaling in human trophoblast development and differentiation. Cell. Mol. Life Sci. 79 (6), 292. 10.1007/s00018-022-04285-3 35562545 PMC9106601

[B24] Ding M. Lu Y. Abbassi S. Li F. Li X. Song Y. (2012). Targeting Runx2 expression in hypertrophic chondrocytes impairs endochondral ossification during early skeletal development. J. Cell. Physiology 227 (10), 3446–3456. 10.1002/jcp.24045 22223437 PMC3359397

[B25] Dong Y. F. Jesse A. M. Kohn A. Gunnell L. M. Honjo T. Zuscik M. J. (2010). RBPjκ-dependent Notch signaling regulates mesenchymal progenitor cell proliferation and differentiation during skeletal development. Development 137 (9), 1461–1471. 10.1242/dev.042911 20335360 PMC2853848

[B26] Duan B. Liu Y. Hu H. Shi F. G. Liu Y. L. Xue H. (2019). Notch1-ADAM8 positive feed-back loop regulates the degradation of chondrogenic extracellular matrix and osteoarthritis progression. Cell Commun. Signal 17 (1), 134. 10.1186/s12964-019-0443-2 31640732 PMC6805603

[B27] Engin F. Lee B. (2010). NOTCHing the bone: insights into multi-functionality. Bone 46 (2), 274–280. 10.1016/j.bone.2009.05.027 19520195 PMC2835535

[B28] Engin F. Yao Z. Yang T. Zhou G. Bertin T. Jiang M. M. (2008). Dimorphic effects of Notch signaling in bone homeostasis. Nat. Med. 14 (3), 299–305. 10.1038/nm1712 18297084 PMC2671578

[B29] Fang S. Liu M. Li L. Zhang F. F. Li Y. Yan Q. (2019). Lymphoid enhancer-binding factor-1 promotes stemness and poor differentiation of hepatocellular carcinoma by directly activating the NOTCH pathway. Oncogene 38 (21), 4061–4074. 10.1038/s41388-019-0704-y 30696957

[B30] Feng J. Zhang Q. Pu F. Zhu Z. Lu K. Lu W. W. (2024). Signalling interaction between β-catenin and other signalling molecules during osteoarthritis development. Cell Prolif. 57 (6), e13600. 10.1111/cpr.13600 PMC11150147 38199244

[B31] Fujimaki R. Tezuka K.-i. (2006). Notch signaling in chondrogenesis. Clin. Calcium 16 (8), 1374–1379. 10.1016/S1937-6448(09)75003-8 16883047

[B32] Fujimaki R. Toyama Y. Hozumi N. Tezuka K. i. (2006). Involvement of notch signaling in initiation of prechondrogenic condensation and nodule formation in limb bud micromass cultures. J. Bone Mineral Metabolism 24 (3), 191–198. 10.1007/s00774-005-0671-y 16622731

[B33] Gao W. Sweeney C. Walsh C. Rooney P. McCormick J. Veale D. J. (2013). Notch signalling pathways mediate synovial angiogenesis in response to vascular endothelial growth factor and angiopoietin 2. Ann. Rheum. Dis. 72 (6), 1080–1088. 10.1136/annrheumdis-2012-201978 23161900 PMC3664379

[B34] Gao J. Fan L. Zhao L. Su Y. (2021). The interaction of Notch and Wnt signaling pathways in vertebrate regeneration. Cell Regen. 10 (1), 11. 10.1186/s13619-020-00072-2 PMC8012441 33791915

[B35] Goldring M. B. Otero M. Tsuchimochi K. Ijiri K. Li Y. (2008). Defining the roles of inflammatory and anabolic cytokines in cartilage metabolism. Ann. Rheum. Dis., 67. iii75-iii82. 10.1136/ard.2008.098764 19022820 PMC3939701

[B36] Goldring M. B. Goldring S. R. (2010). Articular cartilage and subchondral bone in the pathogenesis of osteoarthritis. Ann. N. Y. Acad. Sci. 1192, 230–237. 10.1111/j.1749-6632.2009.05240.x 20392241

[B37] Gopalakrishnan N. Saravanakumar M. Madankumar P. Thiyagu M. Devaraj H. (2014). Colocalization of β-catenin with notch intracellular domain in colon cancer: a possible role of Notch1 signaling in activation of CyclinD1-mediated cell proliferation. Mol. Cell Biochem. 396 (1-2), 281–293. 10.1007/s11010-014-2163-7 25073953

[B38] Grogan S. P. Olee T. Hiraoka K. Lotz M. K. (2008). Repression of chondrogenesis through binding of notch signaling proteins HES-1 and HEY-1 to N-box domains in the COL2A1 enhancer site. Arthritis Rheum. 58 (9), 2754–2763. 10.1002/art.23730 18759300 PMC2786215

[B39] Grogan S. P. Miyaki S. Asahara H. D'Lima D. D. Lotz M. K. (2009). Mesenchymal progenitor cell markers in human articular cartilage: normal distribution and changes in osteoarthritis. Arthritis Res. & Ther. 11 (3), R85. 10.1186/ar2719 19500336 PMC2714136

[B40] Haller R. Schwanbeck R. Martini S. Bernoth K. Kramer J. Just U. (2012). Notch1 signaling regulates chondrogenic lineage determination through Sox9 activation. Cell Death Differ. 19 (3), 461–469. 10.1038/cdd.2011.114 21869831 PMC3278729

[B41] Han J. Zhang J. Zhang X. Luo W. Liu L. Zhu Y. (2024). Emerging role and function of Hippo-YAP/TAZ signaling pathway in musculoskeletal disorders. Stem Cell Res. Ther. 15 (1), 386. 10.1186/s13287-024-04011-9 PMC11520482 39468616

[B42] Hayes A. J. Dowthwaite G. P. Webster S. V. Archer C. W. (2003). The distribution of Notch receptors and their ligands during articular cartilage development. J. Anat. 202 (6), 495–502. 10.1046/j.1469-7580.2003.00185.x 12846471 PMC1571106

[B43] He Y. Li Z. Alexander P. G. Ocasio-Nieves B. D. Yocum L. Lin H. (2020). Pathogenesis of osteoarthritis: risk factors, regulatory pathways in chondrocytes, and experimental models. Biol. (Basel) 9 (8), 194. 10.3390/biology9080194 32751156 PMC7464998

[B44] Heinegård D. Saxne T. (2011). The role of the cartilage matrix in osteoarthritis. Nat. Rev. Rheumatol. 7 (1), 50–56. 10.1038/nrrheum.2010.198 21119607

[B45] Hilton M. J. Tu X. Wu X. Bai S. Zhao H. Kobayashi T. (2008). Notch signaling maintains bone marrow mesenchymal progenitors by suppressing osteoblast differentiation. Nat. Med. 14 (3), 306–314. 10.1038/nm1716 18297083 PMC2740725

[B46] Hoeppner L. H. Secreto F. Jensen E. D. Li X. Kahler R. A. Westendorf J. J. (2009). Runx2 and bone morphogenic protein 2 regulate the expression of an alternative Lef1 transcript during osteoblast maturation. J. Cell Physiol. 221 (2), 480–489. 10.1002/jcp.21879 19650108 PMC3874810

[B47] Hosaka Y. Saito T. Sugita S. Hikata T. Kobayashi H. Fukai A. (2013). Notch signaling in chondrocytes modulates endochondral ossification and osteoarthritis development. Proc. Natl. Acad. Sci. U. S. A. 110 (5), 1875–1880. 10.1073/pnas.1207458110 23319657 PMC3562777

[B48] Hu W. Chen Y. Dou C. Dong S. (2021). Microenvironment in subchondral bone: predominant regulator for the treatment of osteoarthritis. Ann. Rheum. Dis. 80 (4), 413–422. 10.1136/annrheumdis-2020-218089 33158879 PMC7958096

[B49] Huang D. Narayanan N. Cano-Vega M. A. Jia Z. Ajuwon K. M. Kuang S. (2020). Nanoparticle-Mediated inhibition of notch signaling promotes mitochondrial biogenesis and reduces subcutaneous adipose tissue expansion in pigs. iScience 23 (6), 101167. 10.1016/j.isci.2020.101167 32480124 PMC7262558

[B50] Huang Y. Pan W. Bao H. Sun X. Xu C. Ma J. (2025). HSF1 increases EOGT-mediated glycosylation of Notch1 to promote IL-1β-Induced inflammatory injury of chondrocytes. Cartilage, 16 (4), 486–494. 10.1177/19476035241229211 PMC11569509 38366389

[B51] Imayoshi I. Isomura A. Harima Y. Kawaguchi K. Kori H. Miyachi H. (2013). Oscillatory control of factors determining multipotency and fate in mouse neural progenitors. Science 342 (6163), 1203–1208. 10.1126/science.1242366 24179156

[B148] Imhof H. Sulzbacher I. Grampp S. Czerny C. Youssefzadeh S. Kainberger F. (2000). Subchondral bone and cartilage disease: a rediscovered functional unit. Invest. Radiol. 35 (10), 581–588. 10.1097/00004424-200010000-00004 11041152

[B52] Isozaki T. Rabquer B. J. Ruth J. H. Haines G. K. Koch A. E. (2013). ADAM-10 is overexpressed in rheumatoid arthritis synovial tissue and mediates angiogenesis. Arthritis Rheum. 65 (1), 98–108. 10.1002/art.37755 23124962

[B53] Jin G. Z. (2026). Molecular mechanisms of chondrocyte hypertrophy mediated by physical cues and therapeutic strategies in osteoarthritis. Int. J. Mol. Sci. 27 (2), 624. 10.3390/ijms27020624 PMC12840631 41596277

[B149] Kapoor M. Martel-Pelletier J. Lajeunesse D. Pelletier J. P. Fahmi H. (2011). Role of proinflammatory cytokines in the pathophysiology of osteoarthritis. Nat. Rev. Rheumatol. 7 (1), 33–42. 10.1038/nrrheum.2010.196 21119608

[B54] Karlsson C. Brantsing C. Egell S. Lindahl A. (2008). Notch1, Jagged1, and HES5 are abundantly expressed in osteoarthritis. Cells Tissues Organs 188 (3), 287–298. 10.1159/000121610 18354251

[B55] Karlsson C. Brantsing C. Kageyama R. Lindahl A. (2010). HES1 and HES5 are dispensable for cartilage and endochondral bone formation. Cells Tissues Organs 192 (1), 17–27. 10.1159/000280416 20134146

[B57] Kobayashi M. Chijimatsu R. Hart D. A. Hamamoto S. Jacob G. Yano F. (2021). Evidence that TD-198946 enhances the chondrogenic potential of human synovium-derived stem cells through the NOTCH3 signaling pathway. J. Tissue Eng. Regen. Med. 15 (2), 103–115. 10.1002/term.3149 33169924

[B58] Kohn A. Dong Y. Mirando A. J. Jesse A. M. Honjo T. Zuscik M. J. (2012). Cartilage-specific RBPjκ-dependent and -independent notch signals regulate cartilage and bone development. Development 139 (6), 1198–1212. 10.1242/dev.070649 22354840 PMC3283126

[B59] Kohn A. Rutkowski T. P. Liu Z. Mirando A. J. Zuscik M. J. O'Keefe R. J. (2015). Notch signaling controls chondrocyte hypertrophy *via* indirect regulation of Sox9. Bone Res. 3, 15021. 10.1038/boneres.2015.21 26558140 PMC4640428

[B60] Kurenkova A. D. Li L. Usanova A. P. Feng X. Zhou B. Nedorubov A. A. (2023). Notch signaling regulates the chondrogenic potential of both articular chondrocytes and their progenitors during expansion. Stem Cells 41 (6), 658–671. 10.1093/stmcls/sxad031 37085276 PMC10267697

[B61] Kwon C. Cheng P. King I. N. Andersen P. Shenje L. Nigam V. (2011). Notch post-translationally regulates β-catenin protein in stem and progenitor cells. Nat. Cell Biol. 13 (10), 1244–1251. 10.1038/ncb2313 21841793 PMC3187850

[B62] Lefebvre V. Peeters-Joris C. Vaes G. (1990). Modulation by interleukin 1 and tumor necrosis factor alpha of production of collagenase, tissue inhibitor of metalloproteinases and collagen types in differentiated and dedifferentiated articular chondrocytes. Biochim. Biophys. Acta 1052 (3), 366–378. 10.1016/0167-4889(90)90145-4 2162214

[B63] Liao L. Zhang S. Zhao L. Chang X. Han L. Huang J. (2020). Acute synovitis after trauma precedes and is associated with osteoarthritis onset and progression. Int. J. Biol. Sci. 16 (6), 970–980. 10.7150/ijbs.39015 32140066 PMC7053339

[B64] Lin J. He Y. Chen J. Zeng Z. Yang B. Ou Q. (2017). A critical role of transcription factor YY1 in rheumatoid arthritis by regulation of interleukin-6. J. Autoimmun. 77, 67–75. 10.1016/j.jaut.2016.10.008 27829535

[B65] Liu Z. Chen J. Mirando A. J. Wang C. Zuscik M. J. O'Keefe R. J. (2015). A dual role for NOTCH signaling in joint cartilage maintenance and osteoarthritis. Sci. Signal 8 (386), ra71. 10.1126/scisignal.aaa3792 26198357 PMC4607068

[B66] Liu Z. Ren Y. Mirando A. J. Wang C. Zuscik M. J. O'Keefe R. J. (2016). Notch signaling in postnatal joint chondrocytes, but not subchondral osteoblasts, is required for articular cartilage and joint maintenance. Osteoarthr. Cartil. 24 (4), 740–751. 10.1016/j.joca.2015.10.015 26522700 PMC4799757

[B67] Liu Q. Wang J. Chen Y. Zhang Z. Saunders L. Schipani E. (2018). Suppressing mesenchymal stem cell hypertrophy and endochondral ossification in 3D cartilage regeneration with nanofibrous poly(l-lactic acid) scaffold and matrilin-3. Acta Biomater. 76, 29–38. 10.1016/j.actbio.2018.06.027 29940371 PMC6086372

[B68] Liu G. M. Wei J. Xiao W. Xie W. Ru Q. Chen L. (2023). Insights into the Notch signaling pathway in degenerative musculoskeletal disorders: mechanisms and perspectives. Biomed. & Pharmacother. 169, 115884. 10.1016/j.biopha.2023.115884 37981460

[B69] Lolli A. Colella F. De Bari C. van Osch G. J. V. M. (2019). Targeting anti-chondrogenic factors for the stimulation of chondrogenesis: a new paradigm in cartilage repair. J. Orthop. Res. 37 (1), 12–22. 10.1002/jor.24136 30175861

[B70] Muguruma Y. Hozumi K. Warita H. Yahata T. Uno T. Ito M. (2017). Maintenance of bone homeostasis by DLL1-Mediated notch signaling. J. Cell Physiol. 232 (9), 2569–2580. 10.1002/jcp.25647 27735989 PMC5485010

[B71] Majidinia M. Sadeghpour A. Yousefi B. (2018). The roles of signaling pathways in bone repair and regeneration. J. Cell Physiol. 233 (4), 2937–2948. 10.1002/jcp.26042 28590066

[B72] Mamachan M. Sharun K. Banu S. A. Muthu S. Pawde A. M. Abualigah L. (2024). Mesenchymal stem cells for cartilage regeneration: insights into molecular mechanism and therapeutic strategies. Tissue Cell 88, 102380. 10.1016/j.tice.2024.102380 38615643

[B73] Manderfield L. J. Aghajanian H. Engleka K. A. Lim L. Y. Liu F. Jain R. (2015). Hippo signaling is required for notch-dependent smooth muscle differentiation of neural crest. Development 142 (17), 2962–2971. 10.1242/dev.125807 26253400 PMC4582185

[B74] Mapp P. I. Walsh D. A. (2012). Mechanisms and targets of angiogenesis and nerve growth in osteoarthritis. Nat. Rev. Rheumatol. 8 (7), 390–398. 10.1038/nrrheum.2012.80 22641138

[B75] Maudens P. Jordan O. Allémann E. (2018). Recent advances in intra-articular drug delivery systems for osteoarthritis therapy. Drug Discov. Today 23 (10), 1761–1775. 10.1016/j.drudis.2018.05.023 29792929

[B76] Mazor M. Lespessailles E. Best T. M. Ali M. Toumi H. (2022). Gene expression and chondrogenic potential of cartilage cells: osteoarthritis grade differences. Int. J. Mol. Sci. 23 (18), 10610. 10.3390/ijms231810610 36142513 PMC9504485

[B77] Mead T. J. Yutzey K. E. (2009). Notch pathway regulation of chondrocyte differentiation and proliferation during appendicular and axial skeleton development. Proc. Natl. Acad. Sci. U. S. A. 106 (34), 14420–14425. 10.1073/pnas.0902306106 19590010 PMC2732875

[B78] Mirando A. J. Liu Z. Moore T. Lang A. Kohn A. Osinski A. M. (2013). RBP-Jκ-dependent Notch signaling is required for murine articular cartilage and joint maintenance. Arthritis Rheum. 65 (10), 2623–2633. 10.1002/art.38076 23839930 PMC4038327

[B79] Monsalve E. Ruiz-García A. Baladrón V. Ruiz-Hidalgo M. J. Sánchez-Solana B. Rivero S. (2009). Notch1 upregulates LPS‐induced macrophage activation by increasing NF‐κB activity. Eur. J. Immunol. 39 (9), 2556–2570. 10.1002/eji.200838722 19662631

[B81] Mosser D. M. Edwards J. P. (2008). Exploring the full spectrum of macrophage activation. Nat. Rev. Immunol. 8 (12), 958–969. 10.1038/nri2448 19029990 PMC2724991

[B82] Nandagopal N. Santat L. A. LeBon L. Sprinzak D. Bronner M. E. Elowitz M. B. (2018). Dynamic ligand discrimination in the notch signaling pathway. Cell 172 (4), 869–880 e19. 10.1016/j.cell.2018.01.002 PMC6414217 29398116

[B83] Occhetta P. Stüdle C. Barbero A. Martin I. (2016). Learn, simplify and implement: developmental re-engineering strategies for cartilage repai. Swiss Med. Wkly. 146, w14346. 10.4414/smw.2016.14346 27544431

[B84] Oldershaw R. A. Hardingham T. E. (2010). Notch signaling during chondrogenesis of human bone marrow stem cells. Bone 46 (2), 286–293. 10.1016/j.bone.2009.04.242 19406255

[B85] Oldershaw R. A. Tew S. R. Russell A. M. Meade K. Hawkins R. McKay T. R. (2008). Notch signaling through Jagged-1 is necessary to initiate chondrogenesis in human bone marrow stromal cells but must be switched off to complete chondrogenesis. Stem Cells 26 (3), 666–674. 10.1634/stemcells.2007-0806 18192230

[B86] Ortega N. Behonick D. J. Werb Z. (2004). Matrix remodeling during endochondral ossification. Trends Cell Biol. 14 (2), 86–93. 10.1016/j.tcb.2003.12.003 15102440 PMC2779708

[B87] Osipo C. Golde T. E. Osborne B. A. Miele L. A. (2008). Off the beaten pathway: the complex cross talk between Notch and NF-kappaB. Lab. Invest. 88 (1), 11–17. 10.1038/labinvest.3700700 18059366

[B88] Oswald F. Liptay S. Adler G. Schmid R. M. (1998). NF-kappaB2 is a putative target gene of activated Notch-1 *via* RBP-Jkappa. Mol. Cell Biol. 18 (4), 2077–2088. 10.1128/mcb.18.4.2077 9528780 PMC121438

[B89] Pakvasa M. Haravu P. Boachie-Mensah M. Jones A. Coalson E. Liao J. (2021). Notch signaling: its essential roles in bone and craniofacial development. Genes Dis. 8 (1), 8–24. 10.1016/j.gendis.2020.04.006 PMC7859553 33569510

[B90] Palaga T. Buranaruk C. Rengpipat S. Fauq A. H. Golde T. E. Kaufmann S. H. E. (2007). Notch signaling is activated by TLR stimulation and regulates macrophage functions. Eur. J. Immunol. 38 (1), 174–183. 10.1002/eji.200636999 18085664

[B91] Peng Y. Wu S. Li Y. Crane J. L. (2020a). Type H blood vessels in bone modeling and remodeling. Theranostics 10 (1), 426–436. 10.7150/thno.34126 31903130 PMC6929606

[B92] Peng C. Ouyang Y. Lu N. Li N. (2020b). The NF-κB signaling pathway, the Microbiota, and gastrointestinal tumorigenesis: recent advances. Front. Immunol. 11, 1387. 10.3389/fimmu.2020.01387 32695120 PMC7338561

[B93] Proceedings of the National Academy of Sciences (2026).

[B94] Pulai J. I. Chen H. Im H. J. Kumar S. Hanning C. Hegde P. S. (2005). NF-kappa B mediates the stimulation of cytokine and chemokine expression by human articular chondrocytes in response to fibronectin fragments. J. Immunol. 174 (9), 5781–5788. 10.4049/jimmunol.174.9.5781 15843581 PMC2903737

[B95] Rim Y. A. Nam Y. Ju J. H. (2020). The role of chondrocyte hypertrophy and senescence in osteoarthritis initiation and progression. Int. J. Mol. Sci. 21 (7), 2358. 10.3390/ijms21072358 32235300 PMC7177949

[B96] Ross D. A. Kadesch T. (2001). The notch intracellular domain can function as a coactivator for LEF-1. Mol. Cell Biol. 21 (22), 7537–7544. 10.1128/MCB.21.22.7537-7544.2001 11604490 PMC99925

[B97] Rothenfluh D. A. Bermudez H. O'Neil C. P. Hubbell J. A. (2008). Biofunctional polymer nanoparticles for intra-articular targeting and retention in cartilage. Nat. Mater 7 (3), 248–254. 10.1038/nmat2116 18246072

[B98] Ruscitto A. Scarpa V. Morel M. Pylawka S. Shawber C. J. Embree M. C. (2020). Notch regulates fibrocartilage stem cell fate and is upregulated in inflammatory TMJ arthritis. J. Dent. Res. 99 (10), 1174–1181. 10.1177/0022034520924656 32442041 PMC7443994

[B99] Saklatvala J. (1986). Tumour necrosis factor alpha stimulates resorption and inhibits synthesis of proteoglycan in cartilage. Nature 322 (6079), 547–549. 10.1038/322547a0 3736671 PMC7095107

[B100] Schaible H. G. Schmidt R. F. (1985). Effects of an experimental arthritis on the sensory properties of fine articular afferent units. J. Neurophysiol. 54 (5), 1109–1122. 10.1152/jn.1985.54.5.1109 4078610

[B101] Searfoss G. H. Jordan W. H. Calligaro D. O. Galbreath E. J. Schirtzinger L. M. Berridge B. R. (2003). Adipsin, a biomarker of gastrointestinal toxicity mediated by a functional gamma-secretase inhibitor. J. Biol. Chem. 278 (46), 46107–46116. 10.1074/jbc.M307757200 12949072

[B102] Sekine C. Nanki T. Yagita H. (2014). Macrophage-derived delta-like protein 1 enhances interleukin-6 and matrix metalloproteinase 3 production by fibroblast-like synoviocytes in mice with collagen-induced arthritis. Arthritis Rheumatol. 66 (10), 2751–2761. 10.1002/art.38743 24943093

[B103] Shang X. Wang J. Luo Z. Wang Y. Morandi M. M. Marymont J. V. (2016). Notch signaling indirectly promotes chondrocyte hypertrophy *via* regulation of BMP signaling and cell cycle arrest. Sci. Rep. 6, 25594. 10.1038/srep25594 27146698 PMC4857138

[B104] Shin H. M. Minter L. M. Cho O. H. Gottipati S. Fauq A. H. Golde T. E. (2006). Notch1 augments NF-kappaB activity by facilitating its nuclear retention. Embo J. 25 (1), 129–138. 10.1038/sj.emboj.7600902 16319921 PMC1356346

[B105] Si H. Liang M. Cheng J. Shen B. (2019). Effects of cartilage progenitor cells and microRNA-140 on repair of osteoarthritic cartilage injury. Zhongguo Xiu Fu Chong Jian Wai Ke Za Zhi 33 (5), 650–658. 10.7507/1002-1892.201806060 31090363 PMC8337193

[B106] Siebel C. Lendahl U. (2017). Notch signaling in development, tissue homeostasis, and disease. Physiol. Rev. 97 (4), 1235–1294. 10.1152/physrev.00005.2017 28794168

[B146] Sophia Fox A. J. Bedi A. Rodeo S. A. (2009). The basic science of articular cartilage: structure, composition, and function. Sport. Health. 1 (6), 461–468. 10.1177/1941738109350438 23015907 PMC3445147

[B107] Sui Y. Lee J. H. DiMicco M. A. Vanderploeg E. J. Blake S. M. Hung H. H. (2009). Mechanical injury potentiates proteoglycan catabolism induced by interleukin-6 with soluble interleukin-6 receptor and tumor necrosis factor alpha in immature bovine and adult human articular cartilage. Arthritis Rheum. 60 (10), 2985–2996. 10.1002/art.24857 19790045

[B108] Sun H. B. Yokota H. (2002). Reduction of cytokine-induced expression and activity of MMP-1 and MMP-13 by mechanical strain in MH7A rheumatoid synovial cells. Matrix Biol. 21 (3), 263–270. 10.1016/s0945-053x(02)00003-3 12009332

[B109] Sun S. Bay-Jensen A. C. Karsdal M. A. Siebuhr A. S. Zheng Q. Maksymowych W. P. (2014). The active form of MMP-3 is a marker of synovial inflammation and cartilage turnover in inflammatory joint diseases. BMC Musculoskelet. Disord. 15, 93. 10.1186/1471-2474-15-93 24641725 PMC4003863

[B110] Sun J. Luo Z. Wang G. Wang Y. Wang Y. Olmedo M. (2018). Notch ligand Jagged1 promotes mesenchymal stromal cell-based cartilage repair. Exp. Mol. Med. 50 (9), 1–10. 10.1038/s12276-018-0151-9 30242147 PMC6155067

[B111] Suri S. Gill S. E. Massena de Camin S. Wilson D. McWilliams D. F. Walsh D. A. (2007). Neurovascular invasion at the osteochondral junction and in osteophytes in osteoarthritis. Ann. Rheum. Dis. 66 (11), 1423–1428. 10.1136/ard.2006.063354 17446239 PMC2111605

[B112] Swingler T. E. Waters J. G. Davidson R. K. Pennington C. J. Puente X. S. Darrah C. (2009). Degradome expression profiling in human articular cartilage. Arthritis Res. Ther. 11 (3), R96. 10.1186/ar2741 19549314 PMC2714152

[B113] Tian Y. Guo R. Shi B. Chen L. Yang L. Fu Q. (2016). MicroRNA-30a promotes chondrogenic differentiation of mesenchymal stem cells through inhibiting Delta-like 4 expression. Life Sci. 148, 220–228. 10.1016/j.lfs.2016.02.031 26872979

[B114] Toh W. S. Lai R. C. Hui J. H. P. Lim S. K. (2017). MSC exosome as a cell-free MSC therapy for cartilage regeneration: implications for osteoarthritis treatment. Semin. Cell Dev. Biol. 67, 56–64. 10.1016/j.semcdb.2016.11.008 27871993

[B115] Tortorella M. D. Malfait A. M. Deccico C. Arner E. (2001). The role of ADAM-TS4 (aggrecanase-1) and ADAM-TS5 (aggrecanase-2) in a model of cartilage degradation. Osteoarthr. Cartil. 9 (6), 539–552. 10.1053/joca.2001.0427 11520168

[B116] Totaro A. Castellan M. Di Biagio D. Piccolo S. (2018). Crosstalk between YAP/TAZ and notch signaling. Trends Cell Biol. 28 (7), 560–573. 10.1016/j.tcb.2018.03.001 29665979 PMC6992418

[B117] Tschaharganeh D. F. Chen X. Latzko P. Malz M. Gaida M. M. Felix K. (2013). Yes-associated protein up-regulates Jagged-1 and activates the Notch pathway in human hepatocellular carcinoma. Gastroenterology 144 (7), 1530–1542.e12. 10.1053/j.gastro.2013.02.009 23419361 PMC3665638

[B118] Tu X. Chen J. Lim J. Karner C. M. Lee S. Y. Heisig J. (2012). Physiological notch signaling maintains bone homeostasis *via* RBPjk and hey upstream of NFATc1. PLoS Genet. 8 (3), e1002577. 10.1371/journal.pgen.1002577 22457635 PMC3310726

[B56] Venkatesh K. Reddy L. V. K. Abbas S. Mullick M. Moghal E. T. B. Balakrishna J. P. (2017). NOTCH signaling is essential for maturation, self‐renewal, and tri‐differentiation of in vitro derived human neural stem cells. cells. Cell Reprogram 19 (6), 372–383. 10.1089/cell.2017.0009 29035086

[B119] Vernal R. Velásquez E. Gamonal J. Garcia-Sanz J. A. Silva A. Sanz M. (2008). Expression of proinflammatory cytokines in osteoarthritis of the temporomandibular joint. Arch. Oral Biol. 53 (10), 910–915. 10.1016/j.archoralbio.2008.04.004 18508030

[B120] Walsh D. A. McWilliams D. F. Turley M. J. Dixon M. R. Fransès R. E. Mapp P. I. (2010). Angiogenesis and nerve growth factor at the osteochondral junction in rheumatoid arthritis and osteoarthritis. Rheumatol. Oxf. 49 (10), 1852–1861. 10.1093/rheumatology/keq188 20581375 PMC2936950

[B121] Walton B. L. Shattuck-Brandt R. Hamann C. A. Tung V. W. Colazo J. M. Brand D. D. (2025). A programmable arthritis-specific receptor for guided articular cartilage regenerative medicine. Osteoarthr. Cartil. 33 (2), 231–240. 10.1016/j.joca.2024.12.002 PMC12019866 39706287

[B122] Wang L. He C. (2022). Nrf2-mediated anti-inflammatory polarization of macrophages as therapeutic targets for osteoarthritis. Front. Immunol. 13, 967193. 10.3389/fimmu.2022.967193 36032081 PMC9411667

[B123] Wang S. Kawashima N. Sakamoto K. Katsube K. i. Umezawa A. Suda H. (2010). Osteogenic differentiation of mouse mesenchymal progenitor cell, Kusa-A1 is promoted by mammalian transcriptional repressor Rbpj. Biochem. Biophys. Res. Commun. 400 (1), 39–45. 10.1016/j.bbrc.2010.07.133 20691157

[B124] Wang W. Zeng L. Wang Z. m. Zhang S. Rong X. F. Li R. H. (2015). Ginsenoside Rb1 inhibits matrix metalloproteinase 13 through down-regulating notch signaling pathway in osteoarthritis. Exp. Biol. Med. (Maywood) 240 (12), 1614–1621. 10.1177/1535370215587918 26062798 PMC4935351

[B125] Wang W. F. Liu S. Y. Qi Z. F. Lv Z. H. Ding H. R. Zhou W. J. (2020). MiR-145 targeting BNIP3 reduces apoptosis of chondrocytes in osteoarthritis through notch signaling pathway. Eur. Rev. Med. Pharmacol. Sci. 24 (16), 8263–8272. 10.26355/eurrev_202008_22622 32894532

[B126] Wang H. Li M. Wang X. Han J. Zhang X. A. (2025). The Notch signaling pathway in regulating bone and cartilage homeostasis: novel insights into the pathogenesis and therapeutics of osteoarthritis. Cell Commun. Signal. 24 (1), 35. 10.1186/s12964-025-02584-3 41398702 PMC12822221

[B127] Watanabe N. Tezuka Y. Matsuno K. Miyatani S. Morimura N. Yasuda M. (2003). Suppression of differentiation and proliferation of early chondrogenic cells by Notch. J. Bone Mineral Metabolism 21 (6), 344–352. 10.1007/s00774-003-0428-4 14586790

[B128] Wei K. Korsunsky I. Marshall J. L. Gao A. Watts G. F. M. Major T. (2020). Notch signalling drives synovial fibroblast identity and arthritis pathology. Nature 582 (7811), 259–264. 10.1038/s41586-020-2222-z 32499639 PMC7841716

[B129] Wojdasiewicz P. Poniatowski A. Ł Szukiewicz D. (2014). The role of inflammatory and anti-inflammatory cytokines in the pathogenesis of osteoarthritis. Mediat. Inflamm. 2014, 561459. 10.1155/2014/561459 24876674 PMC4021678

[B130] Xiao D. Bi R. Liu X. Mei J. Jiang N. Zhu S. (2019). Notch signaling regulates MMP-13 expression *via* Runx2 in chondrocytes. Sci. Rep. 9 (1), 15596. 10.1038/s41598-019-52125-5 31666602 PMC6821756

[B131] Xu H. Zhu J. Smith S. Foldi J. Zhao B. Chung A. Y. (2012). Notch-RBP-J signaling regulates the transcription factor IRF8 to promote inflammatory macrophage polarization. Nat. Immunol. 13 (7), 642–650. 10.1038/ni.2304 22610140 PMC3513378

[B132] Yamashita S. Andoh M. Ueno-Kudoh H. Sato T. Miyaki S. Asahara H. (2009). Sox9 directly promotes Bapx1 gene expression to repress Runx2 in chondrocytes . Exp. Cell Res. 315 (13), 2231–2240. 10.1016/j.yexcr.2009.03.008 19306868 PMC2696577

[B133] Yang X. Chen L. Xu X. Li C. Huang C. Deng C. X. (2001). TGF-beta/Smad3 signals repress chondrocyte hypertrophic differentiation and are required for maintaining articular cartilage. J. Cell Biol. 153 (1), 35–46. 10.1083/jcb.153.1.35 11285272 PMC2185521

[B134] Yang Z. Tang Y. Zhao Q. Lu H. Xu G. (2018). Down-regulation of microRNA-23b aggravates LPS-induced inflammatory injury in chondrogenic ATDC5 cells by targeting PDCD4. Iran. J. Basic Med. Sci. 21 (5), 529–535. 10.22038/IJBMS.2018.25856.6364 29922435 PMC6000224

[B135] Yu H. T. Gu C. Z. Chen J. Q. (2019). MiR-9 facilitates cartilage regeneration of osteoarthritis in rabbits through regulating Notch signaling pathway. Eur. Rev. Med. Pharmacol. Sci. 23 (12), 5051–5058. 10.26355/eurrev_201906_18168 31298359

[B136] Zack S. R. Alzoubi O. Satoeya N. Singh K. P. Deen S. Nijim W. (2024). Another notch in the Belt of rheumatoid arthritis. Arthritis Rheumatol. 76 (10), 1475–1487. 10.1002/art.42937 38961731 PMC11421962

[B137] Zanotti S. Canalis E. (2016). Notch signaling and the skeleton. Endocr. Rev. 37 (3), 223–253. 10.1210/er.2016-1002 27074349 PMC4890266

[B138] Zhang H. Hilton M. J. Anolik J. H. Welle S. L. Zhao C. Yao Z. (2014). NOTCH inhibits osteoblast formation in inflammatory arthritis *via* noncanonical NF-κB. J. Clin. Invest 124 (7), 3200–3214. 10.1172/JCI68901 24892805 PMC4071381

[B139] Zhang X. He J. Wang W. (2021). Progress in the use of mesenchymal stromal cells for osteoarthritis treatment. Cytotherapy 23 (6), 459–470. 10.1016/j.jcyt.2021.01.008 33736933

[B140] Zhen G. Wen C. Jia X. Li Y. Crane J. L. Mears S. C. (2013). Inhibition of TGF-beta signaling in mesenchymal stem cells of subchondral bone attenuates osteoarthritis. Nat. Med. 19 (6), 704–712. 10.1038/nm.3143 23685840 PMC3676689

[B141] Zheng X. Lin R. Huang S. Zeng Q. Gao X. Liu Y. (2026). DAPT mitigates osteoarthritic cartilage degeneration and enhances articular repair through targeted modulation of the Gucy1a3 and notch signaling axis. Drug Des. Devel Ther. 20, 599941. 10.2147/DDDT.S599941 42157792 PMC13182839

[B142] Zhong L. Huang X. Karperien M. Post J. N. (2016). Correlation between gene expression and osteoarthritis progression in human. Int. J. Mol. Sci. 17 (7), 1126. 10.3390/ijms17071126 27428952 PMC4964500

[B143] Zhou F. Han X. Wang L. Zhang W. Cui J. He Z. (2022a). Associations of osteoclastogenesis and nerve growth in subchondral bone marrow lesions with clinical symptoms in knee osteoarthritis. J. Orthop. Transl. 32, 69–76. 10.1016/j.jot.2021.11.002 34934628 PMC8645426

[B144] Zhou B. Lin W. Long Y. Yang Y. Zhang H. Wu K. (2022b). Notch signaling pathway: architecture, disease, and therapeutics. Signal Transduct. Target Ther. 7 (1), 95. 10.1038/s41392-022-00934-y 35332121 PMC8948217

[B145] Zieba J. T. Chen Y. T. Lee B. H. Bae Y. (2020). Notch signaling in skeletal development, homeostasis and pathogenesis. Biomolecules 10 (2), 332. 10.3390/biom10020332 32092942 PMC7072615

